# Loss of MSH6 or MSH2 sensitizes progressive glioblastoma to radiotherapy

**DOI:** 10.1172/JCI205299

**Published:** 2026-08-25

**Authors:** Vlad Moisoiu, Roxanne Lourman, Frank Szulzewsky, Tobias Kessler, Giulio Collotta, Antonio Porro, Anne Bertolini, Franziska Singer, Patrick J. Cimino, Caroline Hertler, Wolfgang Wick, Guido Reifenberger, Eric C. Holland, Alessandro A. Sartori, Michael Weller, Hans-Georg Wirsching

**Affiliations:** 1 Department of Neurology, University Hospital and University of Zurich, Zurich, Switzerland.; 2 Department of Neurosurgery, Clinical Neurosciences Center and; 3 Huntsman Cancer Institute, University of Utah, Salt Lake City, Utah, USA.; 4 Clinical Cooperation Unit Neurooncology, German Cancer Consortium (DKTK), German Cancer Research Center (DKFZ), Heidelberg, Germany.; 5 Institute of Molecular Cancer Research, University of Zurich, Zurich, Switzerland.; 6 NEXUS Personalized Health, ETH Zurich, Zurich, Switzerland and Swiss Institute of Bioinformatics, Lausanne, Switzerland.; 7 Brain Tumor Institute, Center for Cancer and Immunology Research, Children’s National Research Institute, Washington, DC, USA.; 8 Institute of Neuropathology, Heinrich Heine University and University Hospital Düsseldorf, Düsseldorf, Germany.; 9 German Cancer Consortium (DKTK), partner site Essen/Düsseldorf, Düsseldorf, Germany.; 10 Human Biology Division, Fred Hutchinson Cancer Center, Seattle, WA, USA.; 11 Department of Neurology, Cantonal Hospital Winterthur, Zurich, Switzerland.

**Keywords:** Genetics, Oncology, Brain cancer

## Abstract

<strong>A prime resistance mechanism of glioblastoma to temozolomide is a vulnerability to radiation therapy</strong>

**To the Editor:** Acquired resistance of gliomas to temozolomide is commonly mediated by DNA mismatch repair deficiency (MMR-D) (1). In patients with glioblastoma, MMR-D was associated with epigenetic silencing of *MGMT* and mutated *TP53* (Figure 1A and Supplemental Figure 1A; supplemental material available online with this article; https://doi.org/10.1172/JCI205299DS1). MMR-D and *TP53* mutations were also induced by long-term temozolomide treatment (Supplemental Figure 1B). *MSH6* silencing accelerated growth of untreated glioblastoma stem cells (Figure 1B and Supplemental Figure 1, C–G), but *MSH6* silencing–induced chemoresistance depended on epistatic *TP53* loss (Figure 1C and Supplemental Figure 1H). In patients, inferior postprogression survival was associated with MMR-D, but not with mutated *TP53* (Figure 1D), suggesting that MMR-D accelerates tumor growth beyond chemoresistance.

Genetic murine glioblastoma models reflect key features of human glioblastoma, including histopathologic, cellular, genomic, and spatial gene expression patterns (Figure 1E and Supplemental Figure 1, I–O). Similar to pivotal clinical trials, temozolomide effectiveness was restricted to *Mgmt*-silenced tumors, and *Msh6* silencing restored chemoresistance (Supplemental Figure 2, A–F). Confirming our in vitro observations, *Msh6* silencing accelerated tumor formation independent of *Trp53* silencing (Figure 1F and Supplemental Figure 2, G–J). *Msh6* silencing led to decreased BrdU incorporation, indicating accelerated S-phase progression (Figure 1G) and increased the fraction of cells with focal (G2-phase) or diffuse (M-phase) phospho-histone H3 staining (Supplemental Figure 2, K and L). An increased M:G2 ratio upon *Msh6* silencing indicated inactivation of the G2/M checkpoint (Figure 1H), paralleled by an increase of cCasp3+ apoptotic cells (Supplemental Figure 2M), indicating faster checkpoint progression.

Silencing of Msh2, which heterodimerizes with Msh6 or Msh3 to form the MutSα or MutSβ complexes, also accelerated tumor formation, but this silencing was less pronounced compared with Msh6 silencing (Figure 1I). Silencing of the MutLα components Mlh1 or Pms2 prolonged survival, while silencing of Msh3 did not (Supplemental Figure 2N). These observations reflect intact checkpoint arrest, but inefficient repair upon MutLα loss and a subordinate role of MutSβ in regulating checkpoint initiation or progression.

MMR-D was associated with DNA double-strand break (DSB) repair driver mutations in patients with recurrent glioblastoma and in a pancancer cohort (Supplemental Figure 2, O and P), but whole-exome sequencing of *Msh6*-silenced murine glioblastomas did not identify such an association (Supplemental Figure 2, Q and R), indicating a mutagenesis-independent tumor suppressor function of *Msh6*. Reporter assays demonstrated that distal end joining — which includes non-homologous end joining (NHEJ) — was reduced upon silencing of *MSH6* and, although less pronounced, of *MSH2* (Figure 1J and Supplemental Figure 3, A and B), whereas homologous recombination (HR) was reduced upon *MSH2* silencing only (Figure 1K). Silencing of *MSH6* or *MSH2* also increased postirradiation chromosomal break points (Figure 1, L and M), reduced chromosomal fusions, and favored irradiation-induced accumulation of the DNA damage marker γ-H2ax (Supplemental Figure 3, C and D). Ex vivo irradiation demonstrated that silencing of *Msh6* or *Msh2* led to susceptibility to mitotic catastrophe and DSB, paralleled by radiosensitization (Figure 1, N–R, and Supplemental Figure 3, E–H).

In preclinical trials, *Trp53* silencing rendered tumors resistant to ionizing irradiation, but additional silencing of *Msh6* or *Msh2* induced radiosensitivity (Figure 1, S–U, and Supplemental Figure 3, I–L). Post hoc multivariate analyses of the phase 3 clinical trial NOA-08 (2) demonstrated longer overall survival when radiotherapy was administered upon temozolomide failure, as opposed to the inverse sequence (Figure 1V and Supplemental Figure 3, M and N).

Collectively, our data suggest that temozolomide-induced mutation of *MSH6* or *MSH2* drives disease progression through acceleration of G2/M transition. This noncanonical consequence of *MSH6* or *MSH2* loss causes radiosensitization, advocating biomarker-based patient selection for radiotherapy upon temozolomide failure, similar to observations in endometrial cancer (3). The preferential mutation of MSH6 in progressive glioblastoma is linked to accelerated tumor formation compared with MSH2 loss. This is likely because MSH2-deficient cells are more susceptible to mitotic catastrophe and apoptosis due to simultaneous impairment of HR and NHEJ, which also explains the stronger radiosensitization observed upon MSH2 loss.

Radiation therapy may offer an independent approach to exploit the cancer cell–intrinsic vulnerabilities created by MMR-D, extending beyond recent work that has centered on cancer cell extrinsic immune mechanisms (4), although eradication of glioblastoma will require additional therapeutic means.

The phase 3 clinical trial LEGATO investigates the efficacy of reirradiation in progressive glioblastoma after chemoradiotherapy (NCT05904119). However, the randomized phase 2 trial RTOG-1205 did not identify benefit from reirradiation in glioblastoma (5), suggesting that irradiation-induced radioresistance may counteract MMR-D–associated radiosensitization. This is noteworthy, as most glioblastoma patients receive first-line combined chemoradiotherapy, whereas treatment with sequential temozolomide (at diagnosis) and radiotherapy (at recurrence) is largely restricted to those with unfavorable performance status. Additional preclinical trials in patient-derived glioblastoma models and biomarker-driven, randomized clinical trials are warranted to validate the translatability of our findings.

Detailed methods are available in the Supplemental Materials. Values for all data points in graphs are reported in the Supporting Data Values file.

## Conflict of interest

The authors have declared that no conflict of interest exists.

## Funding support

- Swiss Cancer League (KFS-4870-08-2019).
- OPO Foundation.
- The Desirée and Niels Yde Foundation to HGW and SNSF (320030_219458 to HGW, 31003A_176161 and 310030_208143 to AAS).

## Supplementary Material

Supplemental data

Unedited blot and gel images

Supporting data values

## Figures and Tables

**Figure 1:**
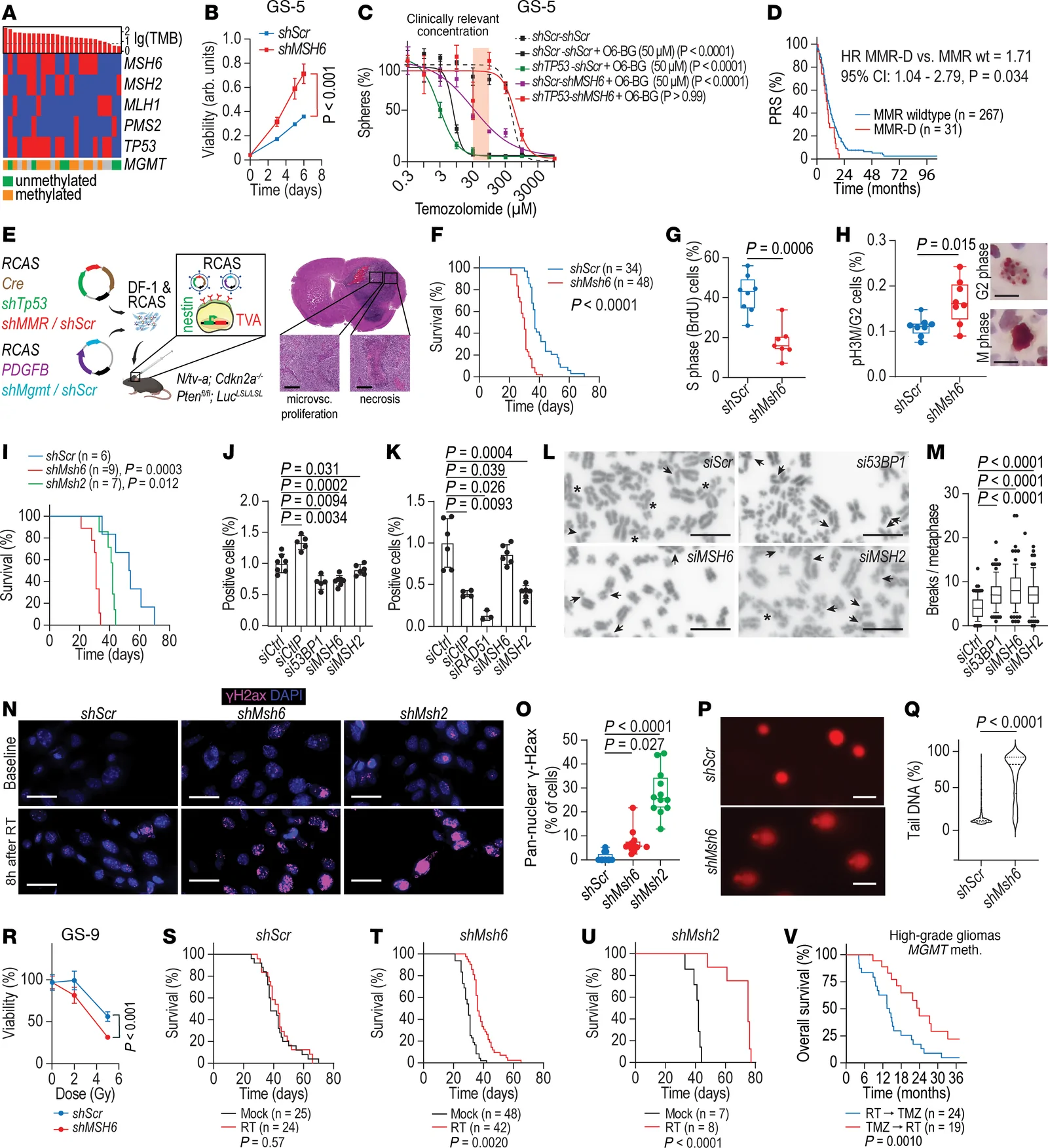
*MSH6 or MSH2* loss accelerates glioblastoma growth and sensitizes to radiotherapy through G2/M checkpoint inhibition. (**A**) MMR mutation frequencies in recurrent glioblastoma. (**B**) MTT viability assay in GS-5 cells; 1-way ANOVA; mean ± SEM. (**C**) Temozolomide (TMZ) concentration-response assay in GS-5 cells; 2-way ANOVA; mean ± SEM. (**D**) Postrecurrence survival (PRS) of patients with glioblastoma; Cox regression accounting for age, *MGMT,* and *TP53* status. (**E**) Schematic of murine glioblastoma modeling; Scale bar: 100 μm. (**F**) Survival of untreated glioblastoma-bearing mice; 2-tailed log-rank. (**G**) BrdU incorporation in murine glioblastomas 24 hours after a single pulse (50 mg/kg); 2-tailed Wilcoxon rank-sum. (**H**) M:G2 ratio based on phospho-histone H3 (pHH3) staining; 2-tailed Wilcoxon rank-sum; Scale bar: 10 μm; (**I**) Survival of untreated glioblastoma-bearing mice; 2-tailed log-rank. (**J** and **K**) Reporter assays quantifying nonhomologous end joining (**J**) and homologous recombination (**K**) in HEK293 cells; 2-tailed *t* test. (**L** and **M**) Metaphase spreads in HEK293 6 hours after 2 Gy irradiation (representative images) (**L**) and quantification of chromosomal breaks; 2-tailed *t* test; Scale bar: 10 μm (**M**). (**N, O**) γ-H2ax staining of murine glioblastoma cells 8 hours after 2 Gy irradiation; Scale bar: 10 μm (**N**), quantification of cells in mitotic catastrophe; 2-tailed Wilcoxon rank-sum (**O**). (**P** and **Q**) Comet assay in GS-9 cells 3 hours after 5 Gy irradiation; Scale bar: 70 μm; 2-tailed Wilcoxon rank-sum. (**R**) MTT viability assay 48 hours after irradiation in GS-9 cells; 1-way ANOVA. (**S**–**U**) Survival of glioblastoma-bearing mice after mock or 5 × 2 Gy head irradiation; 2-tailed log-rank. (**V**) Overall survival of NOA-8 patients by treatment sequence; log-rank. RT, radiotherapy.

## Version 1 08/25/2026

In-Press Preview

## Version 2 10/01/2026

Electronic publication
