# Supplementary material for: Loss of MSH6 or MSH2 sensitizes progressive glioblastoma to radiotherapy
Source: J Clin Invest. 2026 Aug 25;136(19):e205299. doi: 10.1172/JCI205299 (PMC13626850; doi:10.1172/JCI205299)
Supplement: Supplementary file 1 [file jci-136-205299-s216.pdf]

Supplementary Material

Loss of MSH6 or MSH2 sensitizes progressive glioblastoma to radiotherapy

Supplementary material - Online Methods

Table of Contents

Supplementary Figures *4*

List of primary antibodies used in this study. *11*

List of shRNA used in this study. *12*

List of primers used in this study *13*

Patient characteristics in NOA-8. .... *14*

Clinical data *15*

Recurrent glioblastoma cohort 15

NOA-08 cohort 15

Pan-cancer analysis of co-occurrence/mutual exclusivity with MMR-D 16

In vitro assays *16*

Cell culture 16

shRNA-mediated gene silencing in human GS-5 and GS-9 17

Preparation of whole-cell lysates for western blotting 18

Proliferation assay 19

Radiosensitivity assay 20

Temozolomide sensitivity assay 20

Comet assay 21

Double-strand break reporter assays 21

Metaphase spreads 21

In vivo experiments *22*

RCAS vectors design 22

Animal experiments 22

Radiation and chemotherapy treatment 24

Bioluminescence imaging 25

Tumor cell isolation and validation of RCAS/tv-a murine cell lines 25

Cell and tissue imaging *26*

In vivo BrdU staining 26

Quantification of immunohistochemistry stainings 26

Immunofluorescence and quantification of γH2AX foci 27

Sequencing and transcriptomics *28*

Spatial transcriptomics.. 28

Murine tumors collection for RNA sequencing and whole exome sequencing 29

Bioinformatic pipeline for RNA sequencing of murine tumors 29

Bioinformatic pipeline for whole exome sequencing of murine tumors........................ 30

In silico **deconvolution of tumor infiltrating immune cell profile**................................. 30

Statistics *31*

Study approval... *31*

Data availability *31*

Acknowledgments *32*

References *33*

Supplementary Figures

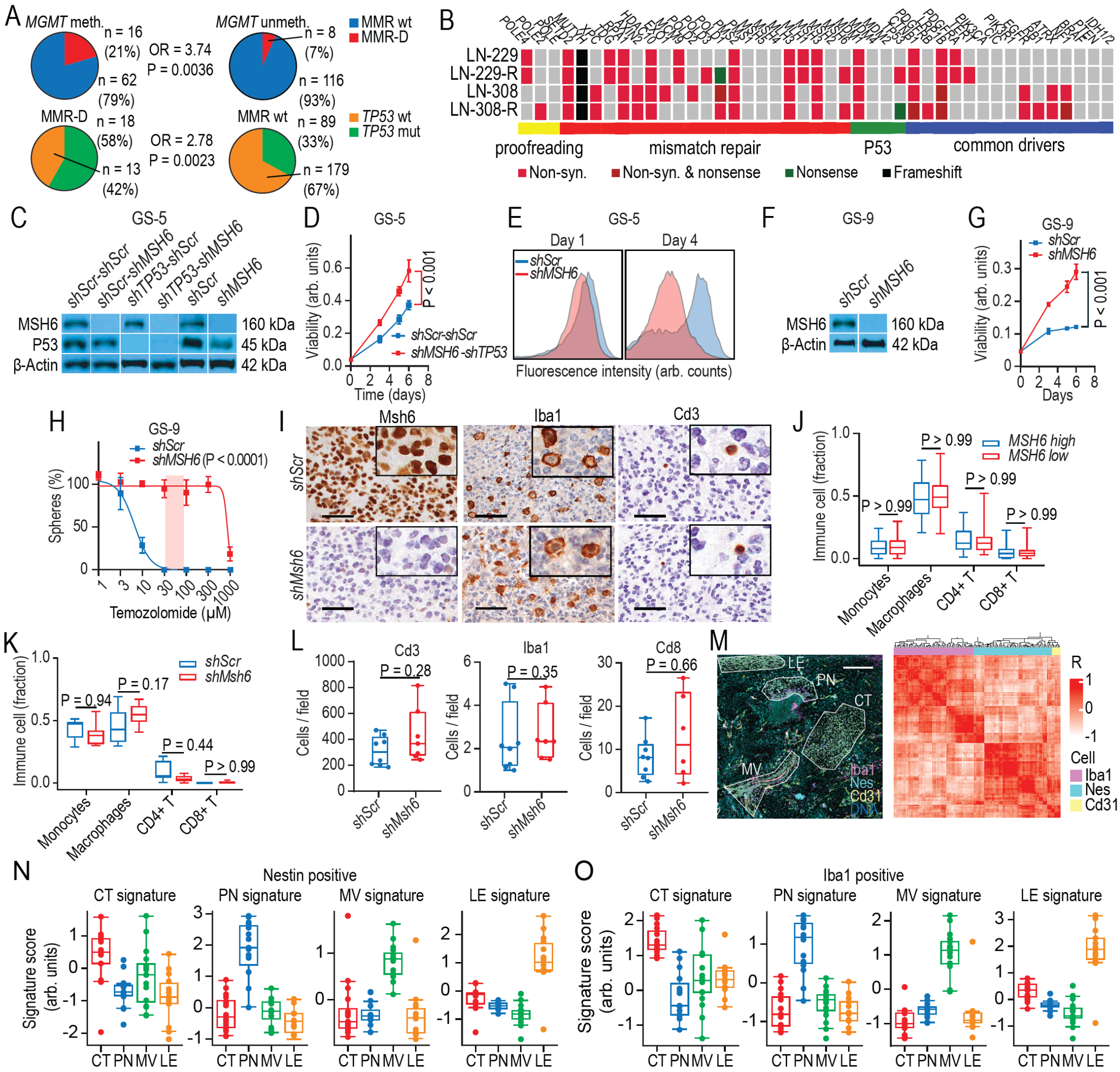

Figure S1. Genetically engineered progressive murine glioblastomas recapitulate the phenotype of human tumors. (**A**) Association of MMR-D with *MGMT* promoter methylation; two-tailed Fisher's exact test (for *MGMT* promoter methylation) and permutation testing (N = 10,000 fold empirical P value) (for MMR-D). (**B**) OncoPrint plot of genomic alterations in native and temozolomide-resistant LN-229 and LN-308 cells. (**C**) Western blot validation of GS-5 cells. **(D)** MTT viability assay in GS-5 cells; two-tailed Wilcoxon rank-sum; mean ± s.e.m. (**E**) CFSE assay

demonstrating the cycling speed of GS-5 cells. (**F**) Western blot validation of GS-9 cells. (**G**) MTT viability assay in GS-9 cells; mean ± s.e.m. (**H**) Temozolomide (TMZ) concentration-response assay in GS-9 cells; two-way ANOVA; mean ± s.e.m. (**I**) Histopathology of genetically engineered progressive glioblastomas in *N/tv-a;Cdkn2a$^{-/-}$;Pten$^{fl/fl}$;Luc$^{LSL/LSL}$* mice utilizing the RCAS/tv-a somatic gene transfer system representing *Msh6*, myeloid cells (Iba-1) and lymphocytes (Cd3); scale bars: 50 µm. (**J**) Tumor-infiltrating immune cell profile of murine glioblastomas deconvoluted from RNA sequencing data by CIBERSORT from (n = 8 samples in each group); two-way ANOVA test with Bonferroni's multiple comparisons test. (**K**) Tumor-infiltrating immune cell profile of human glioblastomas deconvoluted from RNA sequencing data by CIBERSORT (n = 73); two-way ANOVA; the tumors were segregated by median expression of *MSH6*; see Methods for more details. (**L**) quantification of tumor-infiltrating immune cells in murine glioblastoma tumors; two-tailed Wilcoxon rank-sum test; boxes, quartiles; center lines, median; whiskers, absolute range. (**M**) Representative images of regions of interest delineated for spatial transcriptomics and the unsupervised clustering of spatial transcriptomics data for the top 30 most variable genes in the dataset (i.e. highest standard deviation after normalization); the dataset included eight mice in total, of which four mice were transfected with *RCAS-PDGFB-shMgmt* + *RCAS-Cre-shTrp53-shScr* and four mice with *RCAS-PDGFB-shMgmt* + *RCAS-Cre-shTrp53-shMsh6*; CT = cellular tumor, PN = peri-necrotic, MV = microvascular, LE = leading edge; scale bar: 250 µm. (**N**, **O**) The expression levels of signatures specific for histologically-defined niches in murine glioblastoma tumors in Nestin (Nes) positive cells for tumor cells (nestin positive) and tumor-infiltrating macrophages (Iba1 positive); the signatures were derived from the Ivy Glioblastoma

Atlas Project (IvyGAP) by taking the mouse homologs of the human genes; see Methods for more details.

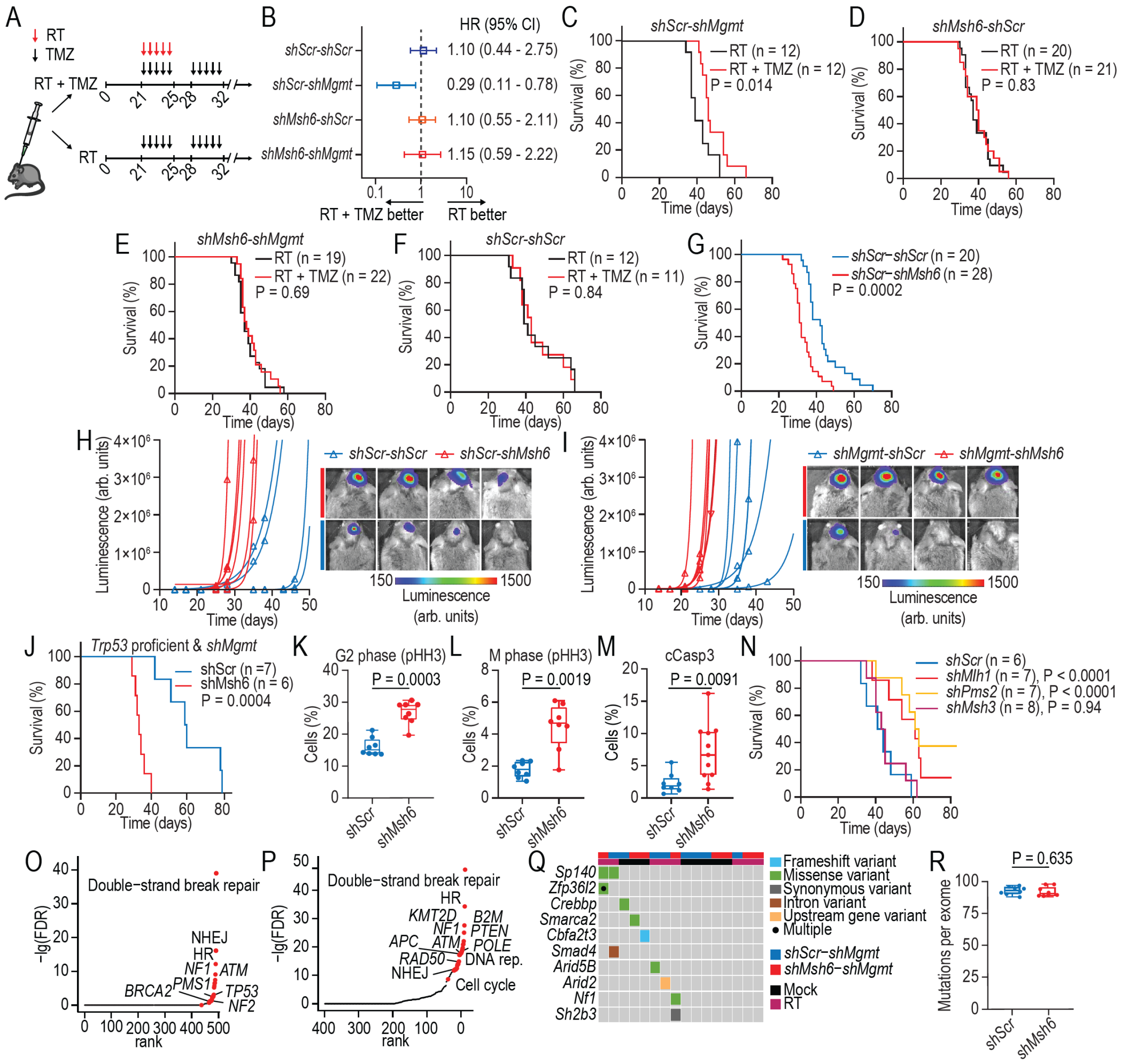

Figure S2. *MSH6* or *MSH2* silencing accelerates tumor formation in genetically engineered progressive murine glioblastomas. (**A**) Schematic representation of the preclinical trial of combined chemoradiotherapy protocol (5 × 2 Gy head irradiation) ± temozolomide (50 mg/kg for five consecutive days) in murine glioblastomas. (**B**) Forest plot of the results of the preclinical trial; two-tailed log-rank test and hazard ratios and 95% confidence intervals. (**C-F**) Survival of mice with indicated genotypes corresponding to the forest plot; two-tailed log-rank test. (**G**) Survival of untreated glioblastoma-bearing mice; two-tailed log-rank test. (**H, I**) tumor growth assessed by in vivo bioluminescence of untreated glioblastoma-bearing mice along with

representative images. (**J**) Survival of untreated glioblastoma-bearing mice; two-tailed log-rank test. (**K, L**) Quantification of focal and diffuse phospho-histone H3 (pHH3) staining in murine glioblastomas; two-tailed Wilcoxon rank-sum; boxes, quartiles; center lines, median; whiskers, absolute range. (**M**) Cleaved caspase-3 staining (apoptosis) in murine glioblastomas; two-tailed Wilcoxon rank-sum; boxes, quartiles; center lines, median; whiskers, absolute range. (**N**) Survival of untreated glioblastoma-bearing mice; two-tailed log-rank test. (**O, P**) Mutations associated with MMR-D in recurrent glioblastoma (n = 299) and pan-cancer cohorts (n = 10,945). (**Q**) OncoPrint plot of mutations in murine glioblastoma tumors (whole-exome sequencing); RT = radiation therapy 5 × 2 Gy. (**R**) Mutations (SNV and indels) in murine glioblastomas from panel (Q); The RT group and mock irradiated mice were pooled; two-tailed Wilcoxon rank-sum; boxes, quartiles; center lines, median; whiskers, absolute range.

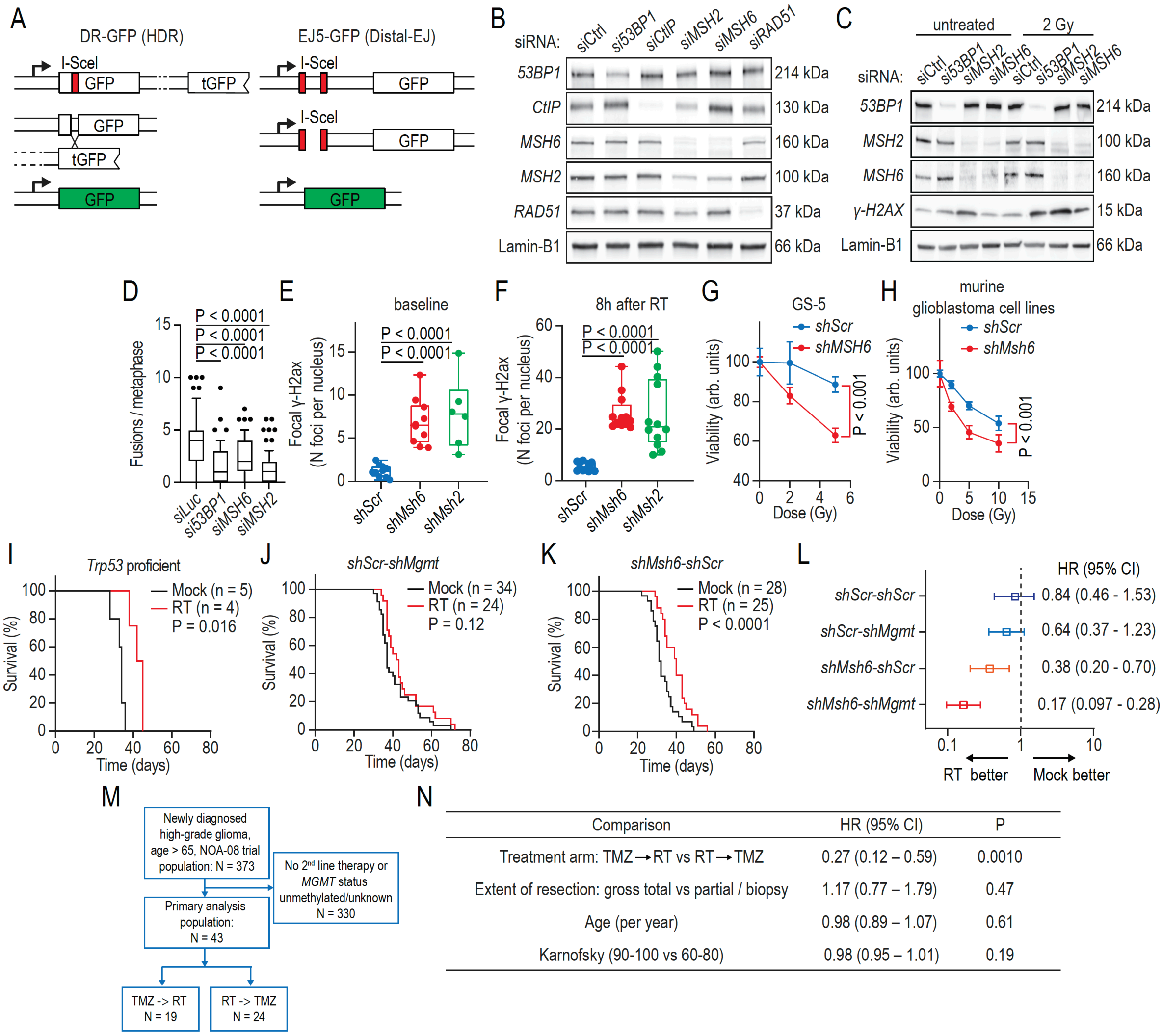

Figure S3. *MSH6* or *MSH2* induces radiosensitivity in murine and human progressive glioblastomas. (**A**) schematic representation of the homologous repair (HR) reporter plasmid (HDR-GFP) and distal end joining (distal-EJ) reporter plasmid (EJ5-GFP). (**B**) Immunoblots corresponding to reporter assays. (**C**) Immunoblots corresponding to metaphase spreads. (**D**) Quantification of fusions per metaphase; boxes, quartiles; bars, mean; whiskers, standard deviation; two-tailed t test. (**E, F**) quantification of the number of γH2ax foci in murine glioblastoma cell lines at baseline (E) and 8 h after radiotherapy (RT, 2 Gy, F); one-way ANOVA test with Bonferroni's correction for multiple testing; boxes, quartiles; bars, mean; whiskers, standard deviation. (**G, H**) MTT viability assay in GS-5 cells (G) and murine glioblastoma cell

lines (H); two-sided Wilcoxon rank-sum; mean ± s.e.m. (**I-K**) Survival of glioblastoma-bearing mice; RT = radiation therapy 5 × 2 Gy; log-rank test. (**L)** Forest plot corresponding to *shMsh6* and *shMgmt* in an *shTrp53* background; log-rank test and hazard ratios and 95% confidence intervals. (**M, N**) CONSORT diagram of NOA-08 trial (M) and the results of the Cox regression (N).

List of primary antibodies used in this study.

Target	Vendor
**Validation of GS-5 and GS-9 by WB**	
MSH6	Cell Signaling Technology #3995
mouse anti-rabbit IgG-HRP	Santa Cruz sc-2357
goat anti-mouse IgG-HRP	BioLegend 405306
mouse anti-β-Actin (C4)-HRP	Santa Cruz sc-47778
**RCAS/tv-a tissue**	
Msh6	Cell Signaling Technology #3995
Cd3	Abcam Ab16669
Cd8	Ebioscience 14-0808-82
Ki67	Abcam Ab16667
pH3	Abcam Ab10543
γH2ax	Thermo Fisher IHC-00059
BrdU	Milipore Sigma MAB3510
**Reporter assay and metaphase spreads western blots**	
MSH6	BD Biosciences, 610919
MSH3	Santa Cruz, H-300
MSH2	Santa Cruz, sc-494
53BP1	Abcam, ab21083
CtIP	Cell Signaling, D76F7
RAD51	Abcam, ab63801
Lamin B1	Abcam, ab16048
γH2AX	Merck, SAB5600038

List of shRNA used in this study.

Target	Sequence
**Short hairpin RNA in GS-5, GS-9 cells**	
shScr	5’-CATATAGCGCACAACTTGACT-3’
shMSH6	5’-GCCAGAAGAATACGAGTTGAA-3’
shTP53	5’-GTCCAGATGAAGCTCCCAGAA-3’
**Short hairpin RNA in RCAS/tv-a mice**	
shMsh6	5’-CCCCGTAATCTACCTGAAGAA-3’
shMsh2	5’-CCCGGCAATCTTTCTCAGTTT -3’
shMlh1	5’-GCAGAGTACATTGTCGAGTTT -3’
shPms2	5’-CCAGGAAGATACCGGATGTAA -3’
shMgmt	5’-CCCCTTTCTTGTTTCTACATTT-3’
shTrp53	5’-GTCCAGATGAAGCTCCCAGAA-3’
**siRNAs in reporter plasmid assays and metaphase spread**	
siCtrl (Luciferase)	5’- CGUACGCGGAAUACUUCGA-3’
si53BP1	5’- CAGGACAGTCTTTCCACGAAT-3’
siCtIP	5’- GCUAAAACAGGAACGAAUC-3’
siMSH2	5’-AACCGGTTGTTGAAAGGC AAA-3’
siMSH6	5’-AAUCUGCAGAGUGUUGUGCUU-3’
siRAD51	5’-AAGGGAATTAGTGAAGCCAAA-3’

List of primers used in this study

Primers	Sequence
**Validation of GS-5, GS-9**	
Human *MSH6* fwd	5’-AGCTTAAAGGATCACGCCATC-3’
Human *MSH6* rev	5’-AAGCACACAATAGGCTTTGCC-3’
Human *TP53* fwd	5’-ACATCTGGCCTTGAAACCAC-3’
Human *TP53* rev	5’-CGAGACCCAGTCTCAAAGAA-3’
Human *ARF1* fwd	5’-GACCACGATCCTCTACAAGC-3’
Human *ARF1* rev	5’-TCCCACACAGTGAAGCTG-3’
**Validation of murine cell lines**	
Mouse *Msh6* fwd	5’-AGGCTGCAGCTGGCAGTGTG-3’
Mouse *Msh6* rev	5’-AGGCCCCTGAACACTGGGCT-3’
Mouse *Msh2* fwd	5’-CTGGTTCGCCAGTATCGAGT-3’
Mouse *Msh2* rev	5’-GATTGCCGGGAGAAGCCTTA-3’
Mouse *Msh3* fwd	5’-GGAAATTACTGCAGGCCAACT-3’
Mouse *Msh3* rev	5’-TGATAAACTCGTACTGTTGGGCA-3’
Mouse *Mlh1* fwd	5’-CCTCCAGGATGTATTTCACCCAG-3’
Mouse *Mlh1* rev	5’-ACGGACCATCTGGTAAGCGTAG-3’
Mouse *Pms2* fwd	5’-CCGACCTAAAGGAACCACAGTC-3’
Mouse *Pms2* rev	5’-ACAGTACGCCTGTAAGACCTGC-3’
Mouse *Arf1* fwd	5’-TCAGCTTCACCGTGTGGGATGT-3’
Mouse *Arf1* rev	5’-CCTCGTTCACACGCTCTCTGTC-3’

Patient characteristics in NOA-8.

Patients with *MGMT* promotor methylated gliomas who were treated in the NOA-08 trial and who crossed over at recurrence. TMZ, temozolomide 200 mg/m^2 on 5/28 days for up to 6 cycles; RT, extended field radiotherapy up to 60 Gy in 30 fractions; KPS, Karnofsy performance score.

	**TMZ→RT (N=19)**	**RT→TMZ (N=24)**
Age: median (range)	73 (66-80)	70.5 (65-81)
Sex: N (%)		
female	10 (53)	12 (50)
male	9 (47)	12 (50)
KPS: median (range)	80 (60-100)	80 (60-100)
Extend of resection: N (%)		
complete	6 (32)	9 (38)
partial	3 (16)	9 (38)
biopsy	10 (53)	6 (25)

Clinical data

Recurrent glioblastoma cohort

The core patient cohort consisted of 299 patients with recurrent Glioblastoma, IDH-wildtype, assembled from three different sources: GENIE 12.0-public, released in July 2022, The GLASS Consortium (1) and Glioma MSKCC database (2). The datasets were downloaded from https://genie.cbioportal.org/ and https://www.cbioportal.org/ on 09.11.2022.

Samples were defined as MMR-D if exhibiting driver mutations in any of the following genes: *MSH6*, *MSH2*, *MLH1*, *PMS2* or if tumors were annotated as hypermutated. Driver mutations were selected as mutations that are oncogenic/likely oncogenic/resistance mutations according to OncoKB (3). For *MGMT* promoter methylation status analysis, the annotation of the recurrent sample was taken into account. In regard to outcomes, MMR-D status was used along with *MGMT* promoter methylation status, *TP53* mutation status and age as input for the Cox regression analysis using post-recurrence survival as readout. Using only *MSH6* and *MSH2* mutations to define MMR-D retained statistical significance (single tailed). Given that MMR-D is associated with an increase in mutation rates, the association between MMR-D and *TP53* pathway mutations was tested empirically by permuting randomly the labels in the *TP53* group 10,000 times and counting the proportion of configurations in which the p-value of Fisher's exact test was smaller for the observed (actual) configuration than the permutated configuration (empirical p value).

NOA-08 cohort

NOA-08 was a randomized, phase 3 trial which assessed temozolomide chemotherapy alone versus radiotherapy alone for malignant astrocytoma in elderly patients(4). After the completion of the trial, subgroups of patients from the temozolomide and radiotherapy arms crossed over, allowing for a comparison of sequential temozolomide followed by radiotherapy vs. radiotherapy followed by temozolomide. Patients with *MGMT* promoter-methylated tumors were included in the analysis.

Pan-cancer analysis of co-occurrence/mutual exclusivity with MMR-D

For the analysis of the association between mutations in the MMR pathway genes and other genes, we analyzed the MSK-IMPACT cohort (5). Pathways were defined as genes related to the same gene ontology (GO) term (6). A pathway was considered as being altered if one or more genes encoding proteins in that particular pathway were mutated. The co-occurrence/mutual exclusivity was tested by Fisher's exact test corrected for multiple testing (false discovery rate).

In vitro assays

Cell culture

The human glioma-initiating cell lines GS-5 and GS-9 were established from freshly dissected glioblastoma tissue (7). Cells were cultivated in neurobasal (NB) medium supplemented with fibroblast growth factor 2 (FGF-2) (20 ng/mL, Gibco, Life Technologies Corporation, #17880953) and epidermal growth factor (EGF) (20 ng/mL; Gibco, Life Technologies Corporation, #AF-100-15), B-27 supplement (20 µL/mL,

Gibco, Life Technologies Corporation, #17504044) and L-glutamine (2 mmol/L, Gibco, Life Technologies Corporation, #25030081). The cell spheres were dissociated into single cells twice per week using Accutase (PAA Laboratories, #A6964-100ML).

The HEK293 cells were cultured in Dulbecco's modified Eagle's medium (DMEM) (Gibco, Life Technologies Corporation, #21068028) supplemented with 10% fetal calf serum (FCS) (Gibco, Life Technologies Corporation, #A5256701) and L-glutamine (2 mmol/L, Gibco, Life Technologies Corporation, #25030081).

Early passage mouse embryonic fibroblast NIH-3T3/tv-a cells were cultivated in DMEM (Gibco, Life Technologies Corporation, #21068028) supplemented with 10% FCS (Gibco, Life Technologies Corporation, #A5256701) and L-glutamine (2 mmol/L, Gibco, Life Technologies Corporation, #25030081).

The chicken embryonic fibroblast DF-1 cells (ATCC reference: CRL-12203) were maintained at low passage number and cultivated in DMEM (Gibco, Life Technologies Corporation, #21068028) supplemented with 10% FCS (Gibco, Life Technologies Corporation, #A5256701) and L-glutamine (4 mmol/L, Gibco, Life Technologies Corporation, #25030081). They were maintained in a 95% humidified incubator at 39°C with 5% CO_2.

Cell lines were regularly tested for mycoplasma contamination using the MycoAlert PLUS Mycoplasma Detection Kit (Lonza, #LT07-703) and were authenticated at the German Collection of Microorganisms and Cell Cultures GmbH (DMSZ Leibniz Institute, Braunschweig, Germany) by short tandem repeat profiling.

<u>shRNA-mediated gene silencing in human GS-5 and GS-9</u>

Briefly, short hairpin RNAs (Sigma Aldrich) were incorporated into the pLKO.1 vector (Addgene) to silence these genes. The shRNA sequences are reported above. Lentiviruses were produced with the $CaCl_2$ method by co-transfection of pLKO.1-shRNA constructs (pLKO.1 shScr vector was used as the control) with the packaging and envelope-expressing plasmids pMD2.G and psPAX2 (r#12259 and #12260, Addgene) into HEK293 cells in antibiotic-free complete DMEM (Gibco, Life Technologies Corporation, #21068028). The medium was exchanged the next day and the viral particles were collected at 48h after the transfection and concentrated using Peg-it precipitation solution (System Biosciences, #LV810A) and then diluted 1:1 with complete antibiotic-free NB medium. Human glioma-initiating cells (GS-9 and GS-5) were seeded at in 6-well plate (Techno Plastic Products AG) at a density of a 10^6 cells/mL then subjected to overnight infection using lentivirus-containing supernatant supplemented with 8 µg/mL Polybrene (Santa Cruz Biotechnology, #28728-55-4). Positively transfected cells were selected using 2 µg/mL Puromycin (Life Technologies, #A1113803) for 3 days. Total RNA of cells for each condition was isolated using the NucleoSpin RNA kit (Macherey-Nagel, #740955.50) to validate gene silencing by reverse transcription-quantitative polymerase chain reaction (RT-qPCR).

Preparation of whole-cell lysates for western blotting

Cells were cultured in NB complete culture medium, washed 2 times with cold PBS (Gibco, Life Technologies Corporation, #10010023) and harvested by centrifugation. Cell pellets were incubated on ice for 30 min. with radioimmunoprecipitation assay (RIPA) lysis and extraction buffer (Thermo Fisher Scientific, #89900), supplemented with 0.66 mM of phenylmethylsulfonyl fluoride, 1:110 diluted Phosphatase Inhibitor

Cocktail 2 and 3 and 1:55 diluted Protease Inhibitor Cocktail (all from Sigma-Aldrich, P5726, P0044, P8340). Pre-cleared whole-cell lysates were mixed and boiled for 5 min at 95°C with Laemmli buffer containing mercaptoethanol (Thermo Fisher Scientific, #J60015.AC). 30 µg of total proteins were loaded and separated using an 8-12% acrylamide SDS-PAGE gel and then transferred onto a methanol-activated polyvinylidene difluoride membrane (PVDF; AmershamTM-Hybond, #10600021) by wet transfer. The membranes were analyzed employing the respective primary antibodies used at concentrations recommended by the manufacturer, and subsequently by the appropriate horseradish peroxidase (HRP)-conjugated secondary antibodies. Proteins were detected by the Pierce enhanced chemiluminescence ECL Western Blotting Substrate (Thermo Fisher Scientific, #32106). The primary antibodies used for immunoblotting analysis are listed above. Protein concentrations were determined using Bicinchoninic acid assay (Thermo Fisher Scientific, #23225) using an Infinite M200 Proplate reader (Tecan).

Proliferation assay

GS-5 and GS-9 were cultivated as spheres, harvested, and dissociated into single cells using Accutase (PAA Laboratories, #A6964-100ML) and seeded in full NB culture medium in 96-well plates at a concentration of 5,000 cells/well. Cell growth rate was followed by MTT assay (Thermo Fisher Scientific, #M6494) every two days by measurement of absorbance at 560 nm with the Infinite M200 Pro plate reader (Tecan).

Proliferation was directly assessed using carboxyfluorescein succinimidyl ester (CFSE, Abcam, #NC0272666). Approximately 3 x 10^6 of GS-5 cells were harvested,

dissociated to single cells using Accutase (PAA Laboratories, #A6964-100ML) and stained with CFSE. Cells were then washed three time with fresh growth medium, seeded in 6-well plates at a density of $5x10^5$ cells/well. Cells were harvested at day 1 and day 4 for FACS analysis of CFSE dye dilution.

<u>Radiosensitivity assay</u>

GS-5 and GS-9 were cultured as spheres, and dissociated into single cells using Accutase (PAA Laboratories, #A6964-100ML) and seeded in full NB culture medium in low adherence 96-well plates at a density of 5,000 cells/well. Murine glioblastoma cell lines were seeded at 5,000 cells/well (see the protocol for isolating the cell lines bellow). The next day, cells were irradiated using RS2000 irradiator (Rad Source Technologies) with different radiation doses.

<u>Temozolomide sensitivity assay</u>

Temozolomide powder (Schering-Plough) was resuspended in 100% dimethylsulfoxide (DMSO) and stock solution aliquots at 200 mM were stored at -80°C for long-term storage. O^6-benzylguanine (O^6-BG) (Sigma-Aldrich, #B2922) stock solutions were prepared in DMSO at a final concentration of 50 mM.

GS-5 and GS-9 glioma-initiating cell lines were cultivated as spheres, dissociated into single cells using Accutase (PAA Laboratories, #A6964-100ML), and were then seeded at a concentration of 250 cells/well in complete NB medium (Gibco, Life Technologies Corporation, #21103049) in 96-well plates. The next day, cells were treated with the drug and incubated at 37°C, 5% CO_2 for 10 days. Spheres were

counted manually at day 10. Spheres smaller than 50 µm were excluded from the analysis.

Comet assay

GS-9 cells were irradiated with 5 Gy and then analyzed by alkaline comet assay 3 h after irradiation(8). The percent of tail DNA was quantified using Comet Score 2.0 (rexhoover.com).

Double-strand break reporter assays

Reporter assays were performed in human embryonic kidney cells (HEK293) (Open BioSystems) using the EJ5-GFP plasmid for distal-EJ(9) and the DR-GFP reporter plasmid for HR(10). 24 h after siRNA transfection (40 nM), the cells were transferred into a 12-well plate at a density of 200,000 cells per well. After 24 h, cells were either mock-transfected or transfected with 0.6 µg of an I-SceI expression plasmid (pCBASce) using 1.2 µl of jetPRIME (Polyplus-transfection, #101000027). 6 h after I-SceI transfection, the medium was replaced, followed by a second transfection with siRNA oligos (15 nM). 48 h after I-SceI transfection, cells were evaluated for green fluorescent protein (GFP) expression by flow cytometry (Attune NxT Flow Cytometer, ThermoFisher Scientific) and analyzed with FlowJo X. Repair efficiency was measured by determining the percentage of GFP-positive cells.

Metaphase spreads

HEK293 cells were transfected with siRNAs (10 nM) for 72 h including a second booster transfection 48 h after first transfection. Cells were exposed to 2 Gy of radiation and cultured for 6 h after irradiation before treatment for 2 hours with Colcemid 100 ng/mL (Gibco, Life Technologies Corporation, #15212012), which arrests cells in metaphase. Cell pellets were resuspended in 5 mL of a hypotonic buffer (75 mM KCl) and incubated for 15 min at 37 °C. Cells were fixed in a methanol/acetic acid (3:1) solution and resuspended in fixative to the appropriate cell density. Cells were spread on glass slides (Menzel-Gläser, ThermoFisher Scientific, #630-1985) and stained with DAPI. Images were acquired at 63x magnification.

***In vivo* experiments**

RCAS vectors design

All the cloning steps to modify inserts were carried out in pENTRmRFP-H1 or pEGH1U6 plasmids and performed by GenScript (GenScript Biotech B.V.). The inserts from the donor pENTR or pEGH1U6 vectors were introduced into the destination plasmid RCAS-DV by gateway cloning using Gateway LR clonase II Enzyme Mix (Invitrogen, #11-791-020). The modified RCAS plasmids were expanded in NEB 10-beta E. coli bacteria (New England Biolabs, #C3019H) under ampicillin selection and purified using the GeneJET Endo-free Plasmid Maxiprep Kit (ThermoFisher Scientific, #K0861).

Animal experiments

The RCAS/tv-a system used in this work has been described previously to model adult gliomas (11). Adult male and female Nestin *(N)/tv-a;Cdkn2a(Ink4a-Arf)*$^{-/-}$*;Pten*$^{fl/fl}$*;Luc*$^{LSL/LSL}$ mice were bred at the animal facility of Laboratory Animal Services Center at the University of Zürich. Our study examined male and female animals, and similar findings are reported for both sexes. Mice were regularly genotyped. At the age of 6-12 weeks, mice were used for the experiments. Murine glioblastomas were generated by stereotactic brain implantation of RCAS-producing DF-1 cells in female and male adult *N/tva; Cdkn2a(Ink4a-Arf)*$^{-/-}$*;Pten*$^{fl/f}$*l;Luc*$^{LSL/LSL}$ mice. Briefly, DF-1 cells were cultured in 6-well plates in complete DMEM (Gibco, Life Technologies Corporation, #21068028) culture media. Seeded DF-1 cells were transfected the next day with the relevant RCAS viral plasmid using FuGENE Transfection reagent (Promega, #E2311) with a FuGENE/DNA ratio of 2:1. Transfected DF-1 cells were incubated at 39°C for 24 h. The cell medium was exchanged for fresh culture medium the next day. RCAS-EGFP was used as a control for efficient transfection and to track in vitro propagation of the RCAS virus. After transfection, DF-1 cells were maintained for at least three passages until the control DF-1 cells transfected with RCAS-EGFP were all infected to ensure a full propagation of the RCAS virus *in vitro*. After validation of efficient virus titration, transfected DF-1 cells were expanded for maximum two additional passages and frozen in separate vials at -80°C for long-term storage. A new vial of DF-1 cells was thawed for each in vivo experiment or set of in vitro experiments. For in vitro experiments, DF-1 cells were regularly passaged with TrypLE Express (Thermo Fisher Scientific, #12604013) twice a week for a maximum of ten passages. Relevant RCAS-producing DF-1 cells were implanted by stereotactic implantation into 6-12 weeks of age *N/tv-a;Cdkn2a(Ink4a-Arf)-/-;Pten*$^{fl/fl}$*;Luc*$^{LSL/LSL}$ mice. Animals were anesthetized by subcutaneous injection of a 3-component anesthetic cocktail (0.05

mg/kg Fentanyl (Kantonsapotheke Zurich), 5 mg/kg midazolam (Roche Pharma AG), 0.5 mg/mL medetomidine (Graeub AG)). Anesthetized mice were immediately injected subcutaneously with 5.7 mg/kg carprofen (Rimadyl, Zoetis GmbH) as analgesia before any surgical procedure. Mice were fixed into the stereotactic frame equipped with a syringe holder/microinjector (Stoelting). A 30G needle was used to make a 1 mm wide hole into the skull at the following coordinates: 2 mm right lateral and 1 mm anterior to the bregma. A total volume of 2 mL of single cell suspension was injected at 3 mm depth and a flow rate of 1000 nL/min using a 10 mL 26-gauge Hamilton syringe (Hamilton Company). A different Hamilton syringe was used for the implantation different cell lines. Mice were then sutured and injected subcutaneously with 1.2 mg/kg naloxone (Kantonsapotheke Zurich). After cell implantation, mice were observed until they developed clear brain tumor related symptoms and/or signs of pain or weight loss of more than 15% below their weight measured at surgery day. Mice were euthanized by CO_2 inhalation, followed by decapitation or organ perfusion.

Radiation and chemotherapy treatment

Mice were anesthetized with subcutaneous injection of anesthetic cocktail. Radiotherapy was performed using RS2000 irradiator (Rad Source Technologies). The head of the mouse was irradiated while the neck and lower body were covered by a lead cover to protect them from full-body irradiation. Mice received intraperitoneally 50 mg/kg temozolomide following radiotherapy. Temozolomide powder was resuspended in saline DPBS solution (Gibco, Life Technologies Corporation, #14190144) containing 0.5% DMSO at a working concentration of 5 mg/mL. Mice received a daily dose of 50

mg/kg for five consecutive days. Similar volume of DPBS + 0.5% DMSO was injected as a control.

Brain tissue was perfused by heart-perfusion with 40 mL of DPBS solution (Gibco, Life Technologies Corporation, #14190144), then fixed with perfusion of 40 mL of 4% Paraformaldehyde (Lucerna Chem, #15733-05). Mouse brain was isolated and further fixed for 24 h in 4% Paraformaldehyde (Lucerna Chem, #15733-05) at 4°C until paraffin-embedding. Immunohistochemistry staining was performed on 5 µm sections of formalin-fixed/paraffin-embedded tissues, on a Discovery XT automated staining processor (Ventana Medical Systems), using the antibodies listed in the Table S2.

Bioluminescence imaging

Bioluminescence imaging was performed using an IVIS Spectrum in vivo imaging system at the Laboratory Animal Services Center at the University of Zürich (ZIRP - Zurich Integrative Rodent Physiology). Luciferin (D-Luciferin, Potassium Salt, ZellBio, #115144-35-9) was injected intraperitoneally under anesthesia (150 mg Luciferin/kg) and images were acquired after 15 min.

Tumor cell isolation and validation of RCAS/tv-a murine cell lines

RCAS/tv-a murine cell lines were established from freshly dissociated murine tumors. Mice were sacrificed when they showed severe signs of intracranial pathology and/or signs of pain. The tumor-bearing hemisphere was collected in ice cold complete DMEM (Gibco, Life Technologies Corporation, #21068028) for transfer. DMEM medium was quickly exchanged for fresh medium containing 100 unit/mL of penicillin

and 100 ug/mL Streptomycin (Pen/Strep, Gibco Life Technologies Corporation, #15140122). The tumor tissue was dissected from the brain hemisphere, cut into thin pieces, and homogenized using a 30G needle. The cell solution was passed through a 100 µm cell strainer (Falcon, #352360) and then incubated at 37°C for 48h in complete DMEM (Gibco, Life Technologies Corporation, #21068028) in cell culture-treated flask. After 48h, cells were washed with ice cold PBS (Gibco, Life Technologies Corporation, #10010023) several times and incubate into culture medium at 37°C. Persistence of gene knockdown in these cells was confirmed by RT-qPCR (Table 3).

Cell and tissue imaging

In vivo BrdU staining

Mice were injected intraperitoneally with 50mg/kg BrdU (Sigma-Aldrich, #19-160) in DPBS (Gibco, Life Technologies Corporation, #14190144) solution when they developed symptoms and were sacrificed 24h after injection for immunostaining. Organs were perfused with PBS (Gibco, Life Technologies Corporation, #10010023), followed by fixation with 4% paraformaldehyde (PFA). Brain and intestine tissues were isolated from the same mouse and fixed with 4% PFA for 24h at 4°C and embedded in paraffin.

Quantification of immunohistochemistry stainings

To quantify positive cells for phospho-H3 and Ki67 stainings, 5-7 images were taken from random regions of each tumor and analyzed using QuPath (12). BrdU positive cells were determined using “Positive cell detection” option in QuPath. For pH3

staining, a set of training images was generated for each class (M phase, G2 phase and negative cells) and used to train a class classifier using the RandomTrees method to distinguish cells in G2 and M phase based on the staining pattern of pH3. For Iba1 staining, a class classifier was trained with a set of training images to improve the detection of positive cell detection based on cell shape.

Immunofluorescence and quantification of γH2AX foci

Adherent RCAS/tv-a tumor cells were seeded at a concentration of 100,000 cells/well and cultured on 12 mm coverslips for 24 h in a 12-well plate. The next day, cells were irradiated using RS2000 irradiator system (Rad Source Technologies) at the indicated doses. Cells were quickly washed and fixed on coverslips using 4% Carson's Phosphate Buffered Paraformaldehyde (Lucerna Chem, #15733-05) diluted with PBS (Gibco, Life Technologies Corporation, #10010023) for 15 min at room temperature at different time points after irradiation. After washing the coverslips with PBS, the cells were permeabilized with 0,25% Triton X-100 (Sigma-Aldrich, X100) in PBS for 20 min at room temperature and blocked with 5% bovine serum albumin (Applichem, #9048-46-8) diluted in PBS (Gibco, Life Technologies Corporation, #10010023). Cells were incubated overnight with the indicated primary antibodies diluted to 1:100 in 5%BSA:PBS (Sigma-Aldrich, #A4737; Gibco, Life Technologies Corporation, #10010023) at room temperature. Coverslips were washed several times with PBS and PBS-Tween 0.1% (Sigma-Aldrich, #A4737 and #P1379) the next day and then incubated with the corresponding secondary antibody for 50 min before being stained for 10 min with DAPI. Coverslips were mounted on microscope slides with Fluorescence mounting medium (Agilent, #S302380-2). Microscope slides were

imaged at the Center for Microscopy of the University of Zürich using a Zeiss Axio Scan.Z1 microscope (Carl Zeiss) with the 40x objective. A total of 10 individual fields of view within each microscope slide were imported and quantified using the CellProfiler. Nuclei were detected based on the DAPI staining intensity and the number of γH2AX foci per nucleus was measured based on the intensity measured in the 647 nm channel. Quantitative results of each field of view were imported in Prism 7 software (GraphPad) for statistical analysis.

Sequencing and transcriptomics

Spatial transcriptomics

GeoMx® DSP (Nanostring, Seattle, USA) was performed on eight tissue samples according to the standard protocol in RCAS/tv-a mice (N/tv-a; Cdkn2a$^{-/-}$ Pten$^{fl/fl}$; Luc$^{LSL/LSL}$) using the following vectors: *RCAS-PDGFB-shMgmt* + *RCAS-Cre-shTp53-shMsh6* (n = 4 mice) or *RCAS-Cre-shTp53-shScr* (n = 4 mice). Immunofluorescence images of the tissues were acquired using the following markers: Nestin for cancer cells, Iba1 for macrophages and Cd31 for endothelial cells. The immunofluorescence images allowed the delineation of the cellular tumor region, perinecrotic region, microvascular proliferation region and leading edge. The regions were manually delineated by P.J.C. After processing, the dataset included 127 regions of interest, of which 31 represented cellular tumor, 39 microvascular proliferation, 30 perinecrotic regions and 27 leading edge. For each region, the GeoMx® DSP yielded count reads for 19,962 genes. The correlation matrix for the regions of interest was calculated using the top 30 genes in terms of standard deviation. The signatures defined by the Ivy Glioblastoma Atlas Project(13) were applied using the R package scalop. Human

genes were converted to their murine orthologues using the *convert_human_to_mouse_symbols* (nichenetr R package, v. 1.1.1).

Murine tumors collection for RNA sequencing and whole exome sequencing

Tumor-bearing brain hemispheres were collected and snap frozen for DNA (whole exome sequencing) and RNA isolation. For whole exome sequencing, we analyzed 16 tumor samples and 6 control brains. Among the 16 tumor samples, eight were represented by tumors induced by *RCAS-PDGFB-shMgmt* + *RCAS-Cre-shTrp53-shScr* and eight with *RCAS-PDGFB-shMgmt* + *RCAS-Cre-shTrp53-shMsh6.* In each group, four tumors received 5X2 Gy on days 21-25 and tumors were collected at endpoint. In the case of RNA sequencing, the genotypes of the tumors were represented by *RCAS-PDGFB-shScr* + *RCAS-Cre-shTrp53-shScr (4 tumors), RCAS-PDGFB-shMgmt* + *RCAS-Cre-shTrp53-shScr (4 tumors), RCAS-PDGFB-shScr* + *RCAS-Cre-shTrp53-shMsh6 (4 tumors), RCAS-PDGFB-shMgmt* + *RCAS-Cre-shTrp53-shMsh6 (4 tumors).* The tumor-free brain hemispheres were collected separately and processed in the same way as the tumor-bearing hemisphere to control for germline mutations. The samples were shipped to Genewiz, which performed the sequencing. For the DNA sequencing, only mutations in the tumor suppressors COSMIC database were considered.

Bioinformatic pipeline for RNA sequencing of murine tumors

Starting from the raw fastq files, adapters were removed and further processed by filtering and trimming the read sequences using the nf-core pipeline(14). For each

sample, reads were mapped to annotated mouse transcripts and the support for each annotated sequence was counted.

The differential expression analysis was focused on the comparison of group "*shScr-shMgmt*" vs."*shMsh6-shMgmt*", aiming to find differentially expressed genes in *Msh6* knockdown condition compared to the vector control, with a *Mgmt* knockdown background of both groups.

Bioinformatic pipeline for whole exome sequencing of murine tumors

Variant entries in VCF files were standardized with bcftools norm (v1.15.1) (15) and further filtered if only a single read supporting the alternative allele was detected, or if overall less than five reads were detected at the variant position, using bcftools filter (v1.15.1). Potential germline variants were filtered by removing all variants that were found in a multiple of the 16 samples, using bcftools (v1.15.1). Variants were annotated using the Ensembl Variant Effect Predictor (VEP) v102 (16). QC measures were gathered with bcftools stats (v1.15.1) and visualized with MultiQC (v1.17) (17).

In silico deconvolution of tumor infiltrating immune cell profile

To deconvolute the tumor infiltrating immune cells, the murine immune cell signatures from Chen et al. (18) were used as input for the CIBERSORT algorithm (19) and applied on the RNA sequencing data (tpm counts) from RCAS/tv-a tumors. For human glioblastoma samples, the dataset from Wang et al. (20) was used.

Statistics

A P value less than 0.05 was considered significant. All statistical tests were two-tailed. The test used for each comparison is indicated in the figure captions. The statistical tests for boxplots are two-sided Wilcoxon rank-sum or one-way ANOVA, unless otherwise stated and log-rank for survival curves. Boxplots: boxes, quartiles; center lines, median; whiskers, absolute range. For the two-way ANOVA test, Bonferroni's multiple comparisons test was used.

Study approval

All animal experiments were performed in accordance with the general guidelines of the Swiss federal law on animal protection and with the protocols approved by the Institutional Animal Care and Use Committee of the cantonal veterinary office (license numbers ZH098/2018 and ZH109/2020).

Data availability

The RNA sequencing data of murine tumors and the whole exome sequencing data of parental and temozolomide-resistant cell lines are available in the Sequence Read Archive (SRA) under accession number PRJNA1479785. The raw flow cytometry (FACS) data are available at Zenodo with DOI 10.5281/zenodo.20785964. The raw data of the NOA-08 trial are available from the corresponding author upon request. The datasets required to reproduce the figures are provided in the Supporting Data Values XLS file.

Acknowledgments

We are thankful for the support of the Department of Pathology at University Hospital Zürich (Susanne Dettwiler and Fabiola Prutek) and the Center for Microscopy and Image Analysis of University of Zürich.

References

1. Barthel FP, Johnson KC, Varn FS, Moskalik AD, Tanner G, Kocakavuk E, et al. Longitudinal molecular trajectories of diffuse glioma in adults. Nature. 2019 Dec;576(7785):112–20. doi:10.1038/s41586-019-1775-1 PubMed PMID: 31748746; PubMed Central PMCID: PMC6897368.

2. Jonsson P, Lin AL, Young RJ, DiStefano NM, Hyman DM, Li BT, et al. Genomic Correlates of Disease Progression and Treatment Response in Prospectively Characterized Gliomas. Clin Cancer Res. 2019 Sep 15;25(18):5537–47. doi:10.1158/1078-0432.CCR-19-0032 PubMed PMID: 31263031; PubMed Central PMCID: PMC6753053.

3. Chakravarty D, Gao J, Phillips SM, Kundra R, Zhang H, Wang J, et al. OncoKB: A Precision Oncology Knowledge Base. JCO Precis Oncol. 2017 Jul;2017. doi:10.1200/PO.17.00011 PubMed PMID: 28890946; PubMed Central PMCID: PMC5586540.

4. Wick W, Platten M, Meisner C, Felsberg J, Tabatabai G, Simon M, et al. Temozolomide chemotherapy alone versus radiotherapy alone for malignant astrocytoma in the elderly: the NOA-08 randomised, phase 3 trial. Lancet Oncol. 2012 Jul;13(7):707–15. doi:10.1016/S1470-2045(12)70164-X PubMed PMID: 22578793.

5. Zehir A, Benayed R, Shah RH, Syed A, Middha S, Kim HR, et al. Mutational landscape of metastatic cancer revealed from prospective clinical sequencing of 10,000 patients. Nat Med. 2017 Jun;23(6):703–13. doi:10.1038/nm.4333 PubMed PMID: 28481359; PubMed Central PMCID: PMC5461196.

6. Harris MA, Clark J, Ireland A, Lomax J, Ashburner M, Foulger R, et al. The Gene Ontology (GO) database and informatics resource. Nucleic Acids Res. 2004 Jan 1;32(Database issue):D258-261. doi:10.1093/nar/gkh036 PubMed PMID: 14681407; PubMed Central PMCID: PMC308770.

7. Günther HS, Schmidt NO, Phillips HS, Kemming D, Kharbanda S, Soriano R, et al. Glioblastoma-derived stem cell-enriched cultures form distinct subgroups according to molecular and phenotypic criteria. Oncogene. 2008 May 1;27(20):2897–909. doi:10.1038/sj.onc.1210949 PubMed PMID: 18037961.

8. Collins A, Møller P, Gajski G, Vodenková S, Abdulwahed A, Anderson D, et al. Measuring DNA modifications with the comet assay: a compendium of protocols. Nat Protoc. 2023 Mar;18(3):929–89. doi:10.1038/s41596-022-00754-y PubMed PMID: 36707722; PubMed Central PMCID: PMC10281087.

9. Bennardo N, Cheng A, Huang N, Stark JM. Alternative-NHEJ is a mechanistically distinct pathway of mammalian chromosome break repair. PLoS Genet. 2008 Jun 27;4(6):e1000110. doi:10.1371/journal.pgen.1000110 PubMed PMID: 18584027; PubMed Central PMCID: PMC2430616.

10. Bennardo N, Gunn A, Cheng A, Hasty P, Stark JM. Limiting the persistence of a chromosome break diminishes its mutagenic potential. PLoS Genet. 2009

Oct;5(10):e1000683. doi:10.1371/journal.pgen.1000683 PubMed PMID: 19834534; PubMed Central PMCID: PMC2752804.

11. Ozawa T, Riester M, Cheng YK, Huse JT, Squatrito M, Helmy K, et al. Most human non-GCIMP glioblastoma subtypes evolve from a common proneural-like precursor glioma. Cancer Cell. 2014 Aug 11;26(2):288–300. doi:10.1016/j.ccr.2014.06.005 PubMed PMID: 25117714; PubMed Central PMCID: PMC4143139.

12. Bankhead P, Loughrey MB, Fernández JA, Dombrowski Y, McArt DG, Dunne PD, et al. QuPath: Open source software for digital pathology image analysis. Sci Rep. 2017 Dec 4;7(1):16878. doi:10.1038/s41598-017-17204-5 PubMed PMID: 29203879; PubMed Central PMCID: PMC5715110.

13. Puchalski RB, Shah N, Miller J, Dalley R, Nomura SR, Yoon JG, et al. An anatomic transcriptional atlas of human glioblastoma. Science. 2018 May 11;360(6389):660–3. doi:10.1126/science.aaf2666 PubMed PMID: 29748285; PubMed Central PMCID: PMC6414061.

14. Ewels PA, Peltzer A, Fillinger S, Patel H, Alneberg J, Wilm A, et al. The nf-core framework for community-curated bioinformatics pipelines. Nat Biotechnol. 2020 Mar;38(3):276–8. doi:10.1038/s41587-020-0439-x PubMed PMID: 32055031.

15. Danecek P, Bonfield JK, Liddle J, Marshall J, Ohan V, Pollard MO, et al. Twelve years of SAMtools and BCFtools. Gigascience. 2021 Feb 16;10(2):giab008. doi:10.1093/gigascience/giab008 PubMed PMID: 33590861; PubMed Central PMCID: PMC7931819.

16. McLaren W, Gil L, Hunt SE, Riat HS, Ritchie GRS, Thormann A, et al. The Ensembl Variant Effect Predictor. Genome Biol. 2016 Jun 6;17(1):122. doi:10.1186/s13059-016-0974-4 PubMed PMID: 27268795; PubMed Central PMCID: PMC4893825.

17. Ewels P, Magnusson M, Lundin S, Käller M. MultiQC: summarize analysis results for multiple tools and samples in a single report. Bioinformatics. 2016 Oct 1;32(19):3047–8. doi:10.1093/bioinformatics/btw354 PubMed PMID: 27312411; PubMed Central PMCID: PMC5039924.

18. Chen Z, Quan L, Huang A, Zhao Q, Yuan Y, Yuan X, et al. seq-ImmuCC: Cell-Centric View of Tissue Transcriptome Measuring Cellular Compositions of Immune Microenvironment From Mouse RNA-Seq Data. Front Immunol. 2018;9:1286. doi:10.3389/fimmu.2018.01286 PubMed PMID: 29922297; PubMed Central PMCID: PMC5996037.

19. Newman AM, Liu CL, Green MR, Gentles AJ, Feng W, Xu Y, et al. Robust enumeration of cell subsets from tissue expression profiles. Nat Methods. 2015 May;12(5):453–7. doi:10.1038/nmeth.3337 PubMed PMID: 25822800; PubMed Central PMCID: PMC4739640.

20. Wang J, Cazzato E, Ladewig E, Frattini V, Rosenbloom DIS, Zairis S, et al. Clonal evolution of glioblastoma under therapy. Nat Genet. 2016 Jul;48(7):768–76.

doi:10.1038/ng.3590 PubMed PMID: 27270107; PubMed Central PMCID: PMC5627776.
