# Supplementary material for: Loss of MSH6 or MSH2 sensitizes progressive glioblastoma to radiotherapy
Source: J Clin Invest. 2026 Aug 25;136(19):e205299. doi: 10.1172/JCI205299 (PMC13626850; doi:10.1172/JCI205299)

Raw western blots Figure S1C - MSH6

Mouse Antibody #610918, GTBP
1:1000, 5% BSA
Transfer time : 1h30-1h45
MSH6: 160 kDa
10% acrylamide

Samples :
1. GS-5 shSCR DMSO/DMSO
2. GS-5 shSCR DMSO/TMZ
3. GS-5 shSCR O6-BG/TMZ
4. GS-5 shMSH6 - shSCR DMSO/DMSO
5. GS-5 shMSH6 - shSCR DMSO/TMZ
6. GS-5 shMSH6 - shSCR O6-BG/TMZ
7. GS-5 shMSH6 - shTP53 DMSO/DMSO
8. " O6-BG/DMSO
9. " DMSO/TMZ
10. " O6-BG/TMZ
11. GS-9 shMSH6 (negative control)

① ② ③ . . . . . .

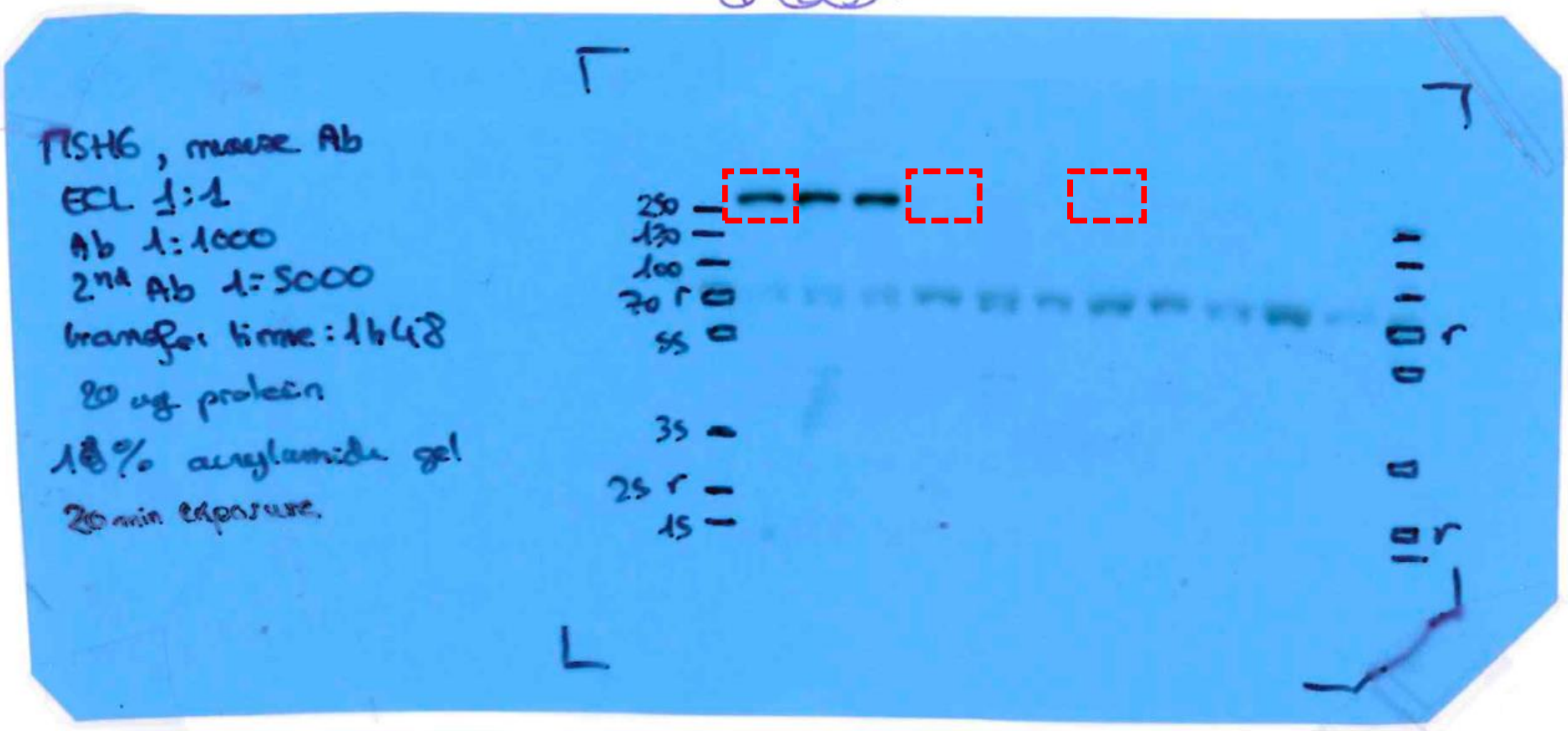

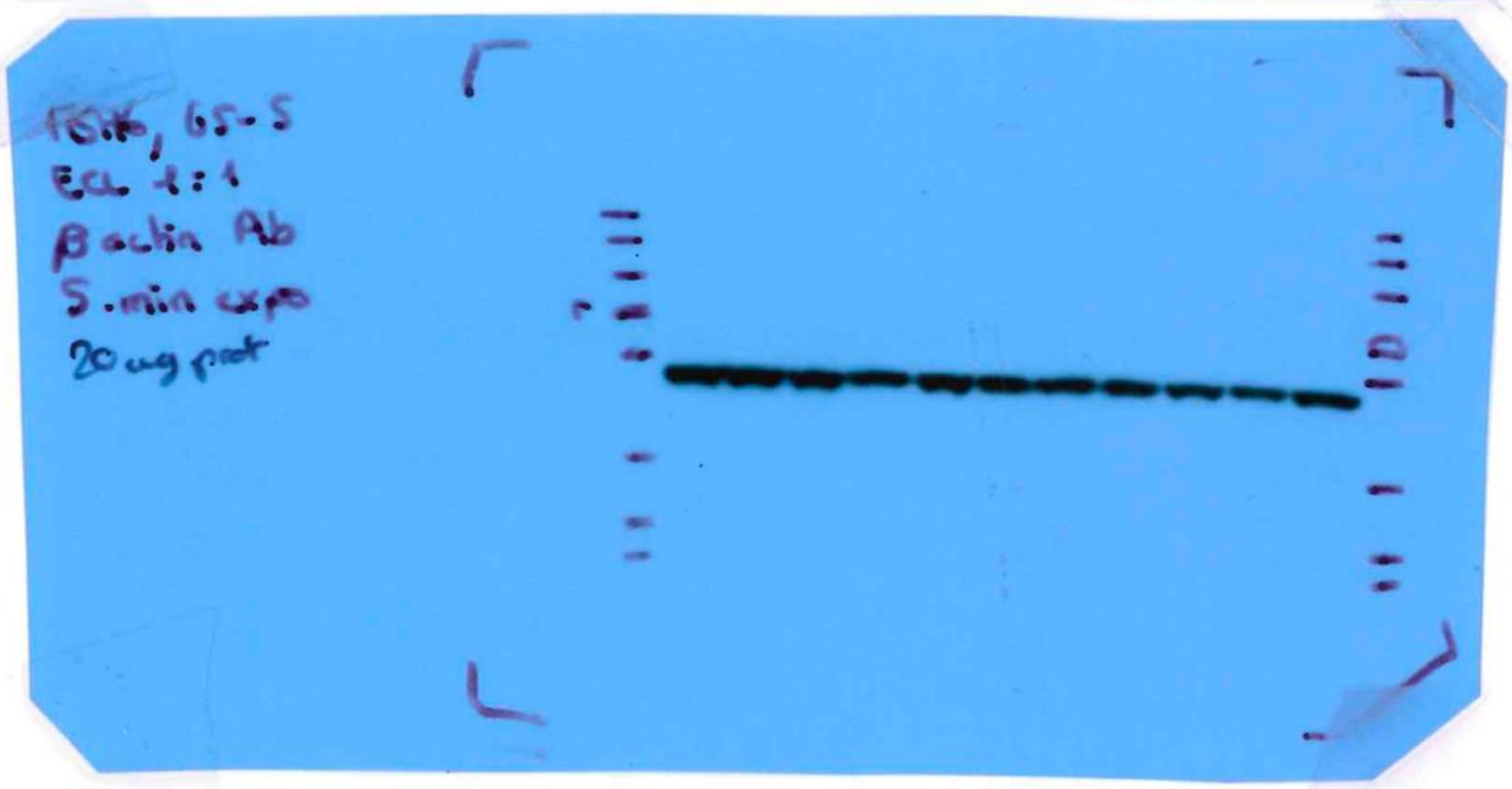

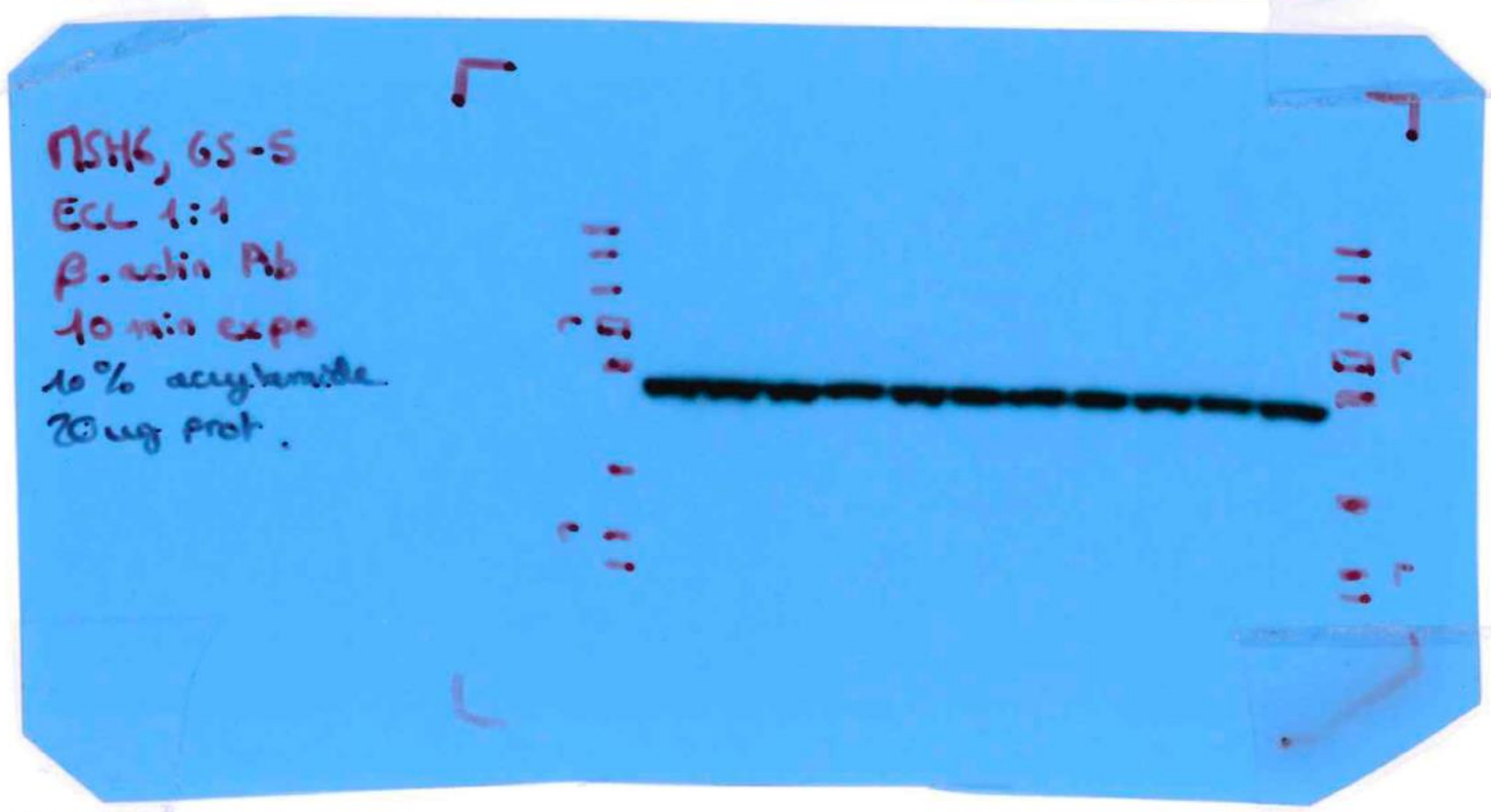

Raw western blots Figure S1C TP53 & β actin

Mouse Antibody D0-1
sc-126
1:1000
#476 U19, Box 8, B7
5% skim milk

P53: 53 kDa
10% acrylamide gel

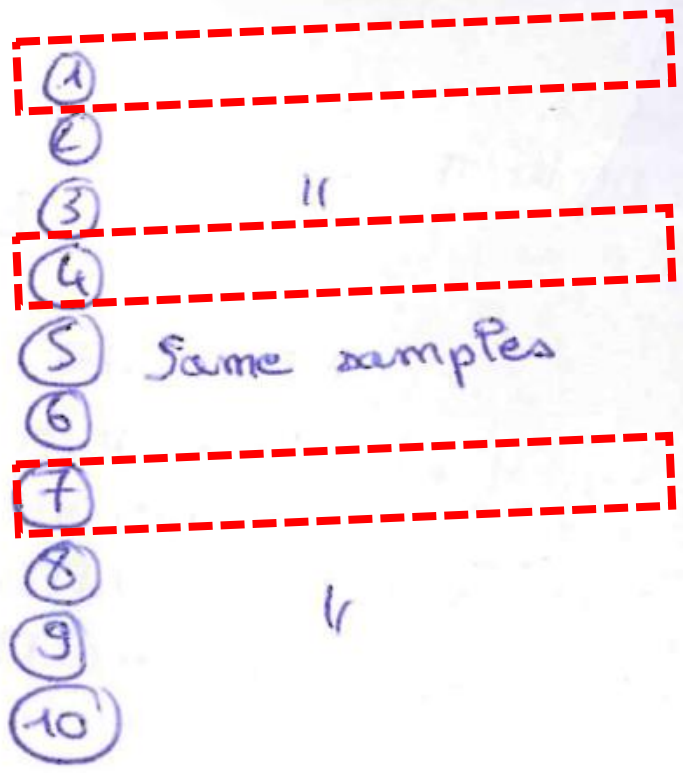

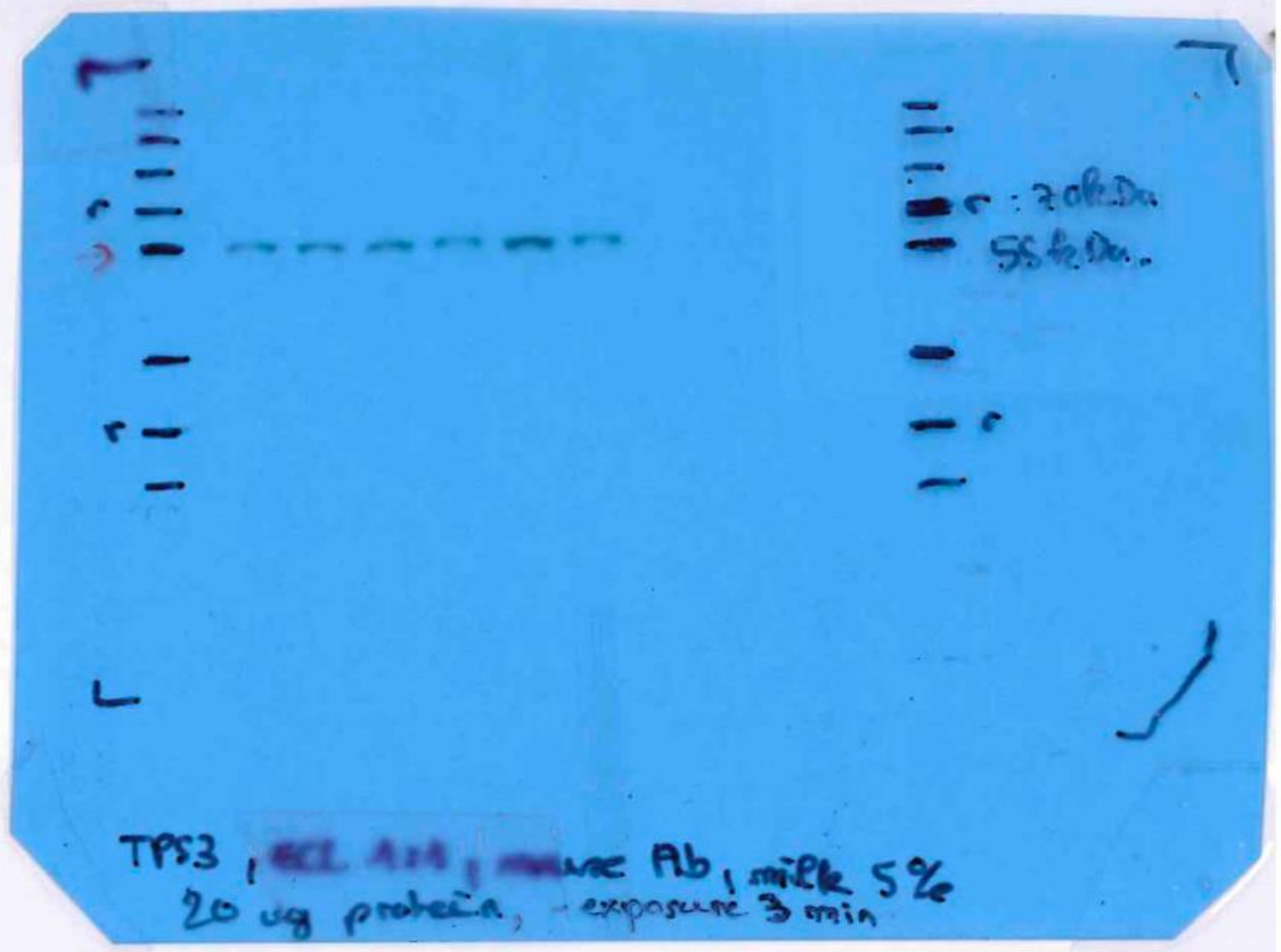

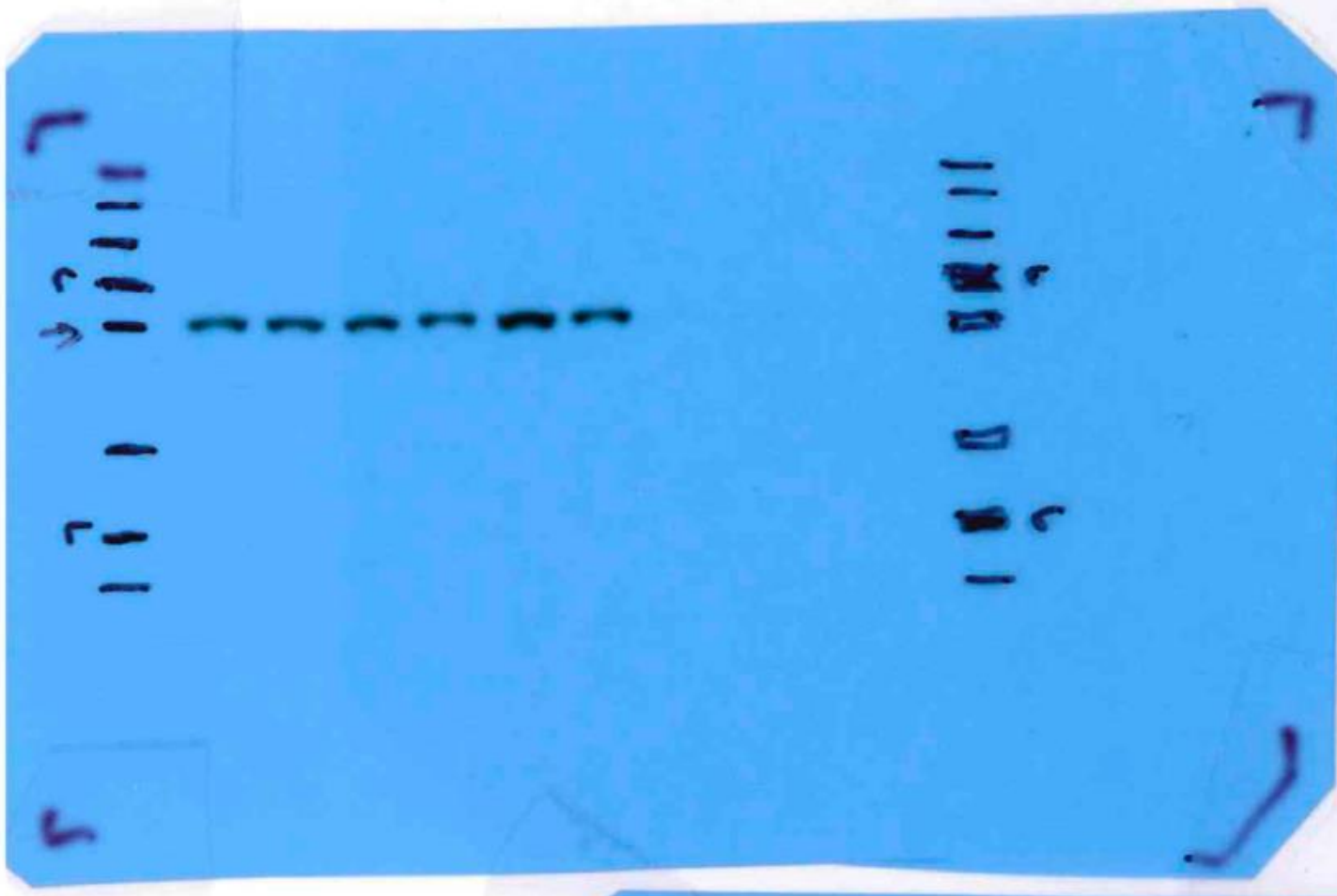

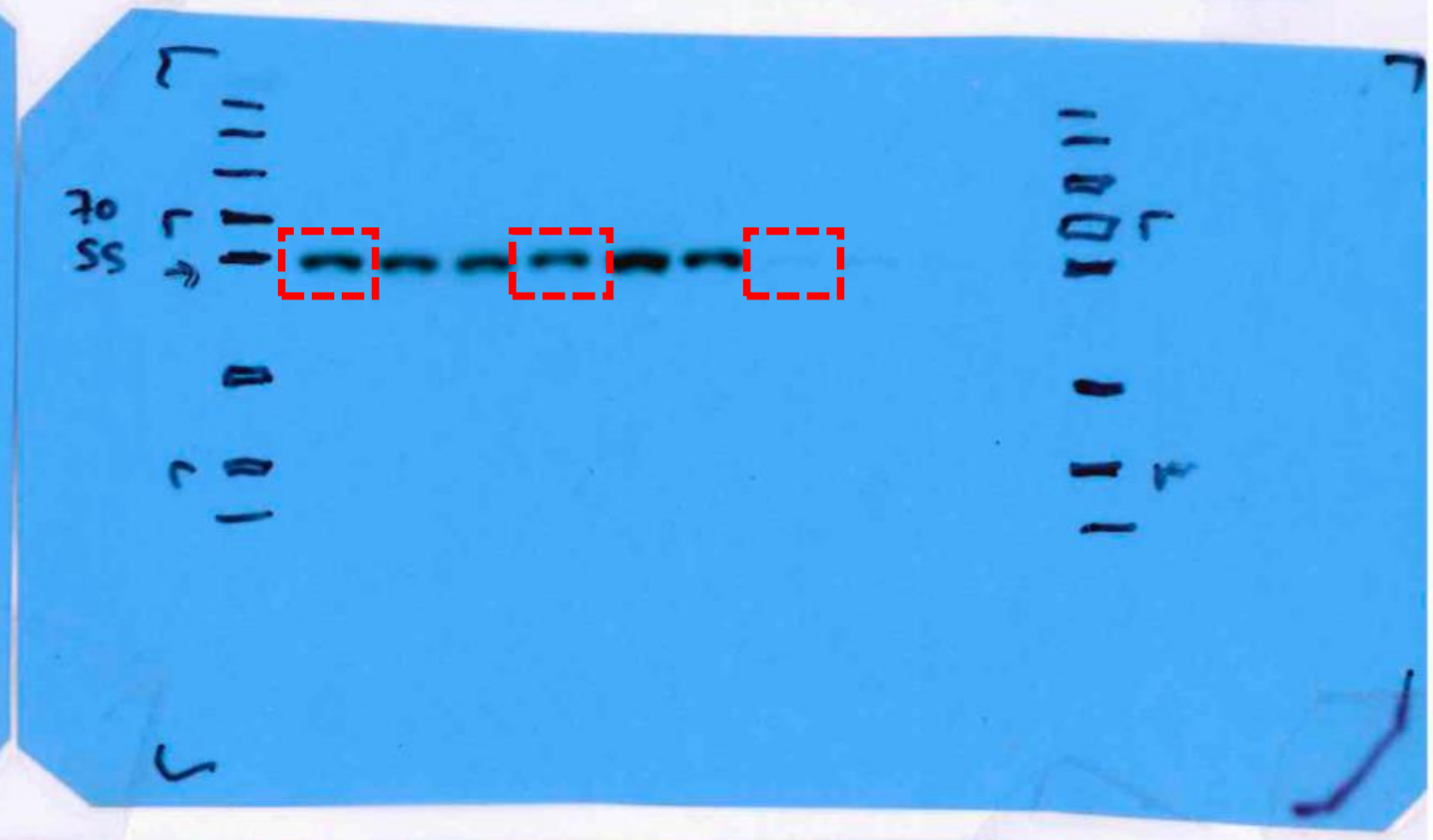

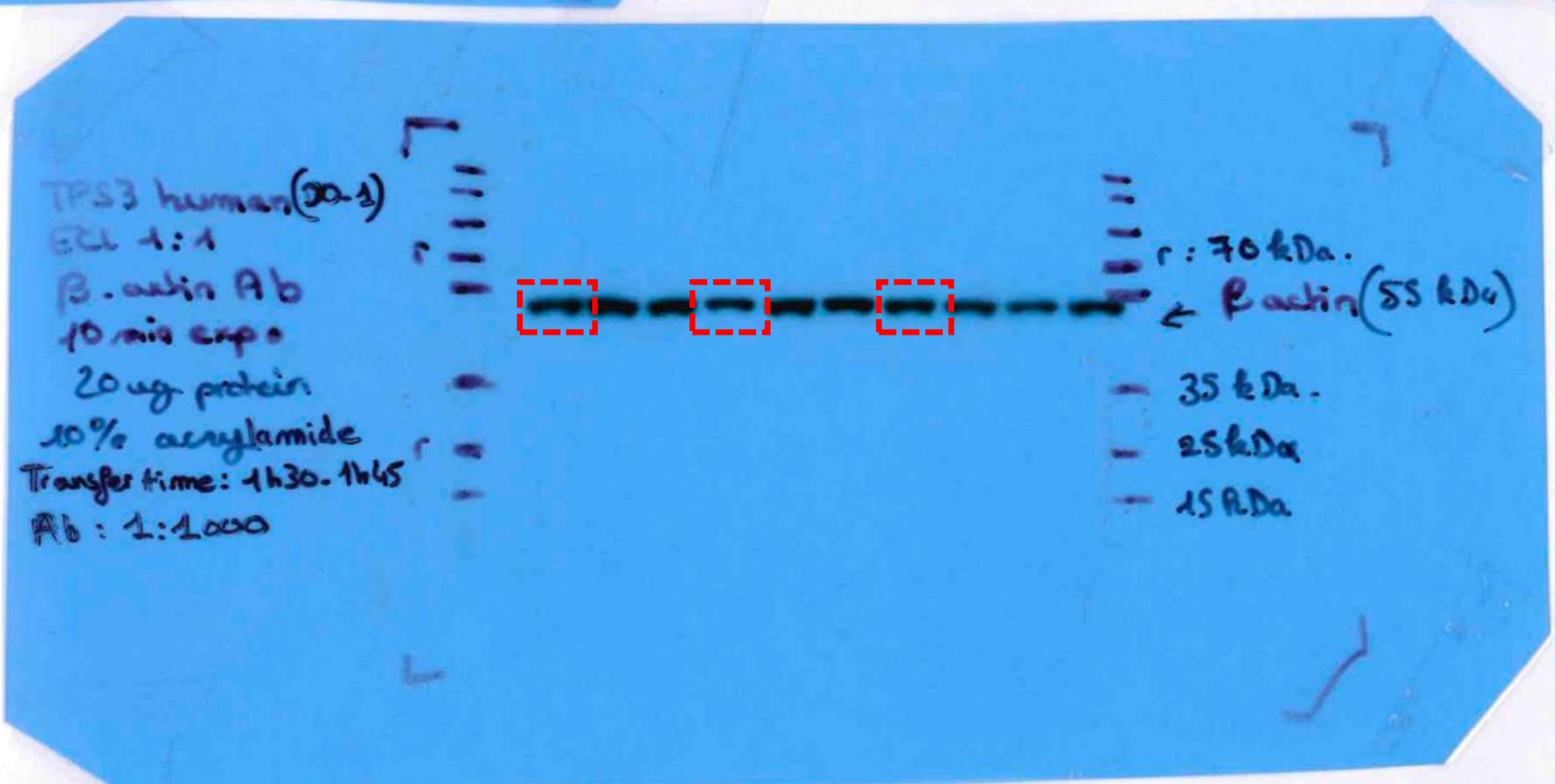

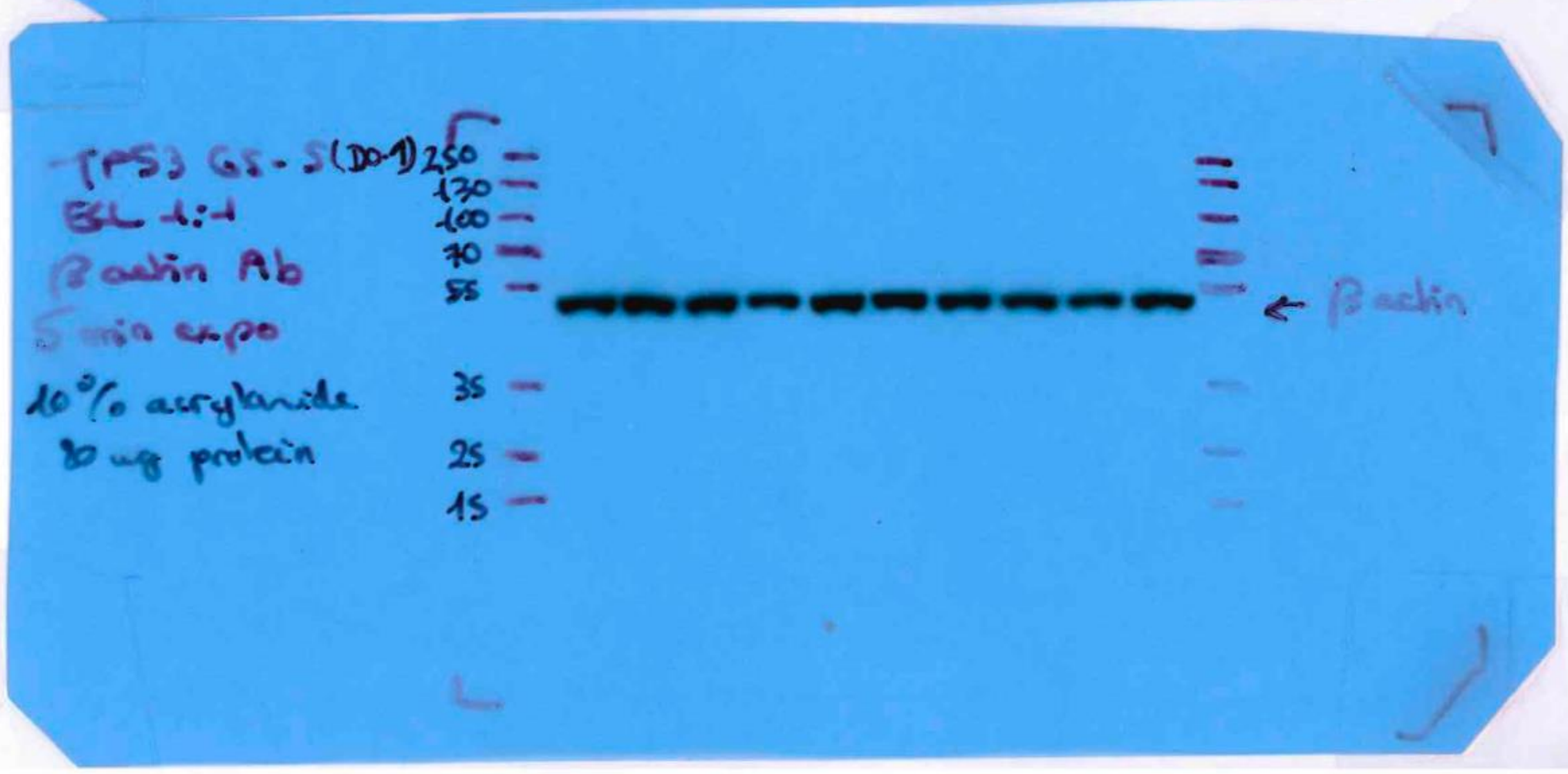

Raw western blots Figure S1C MSH6, TP53 & β actin

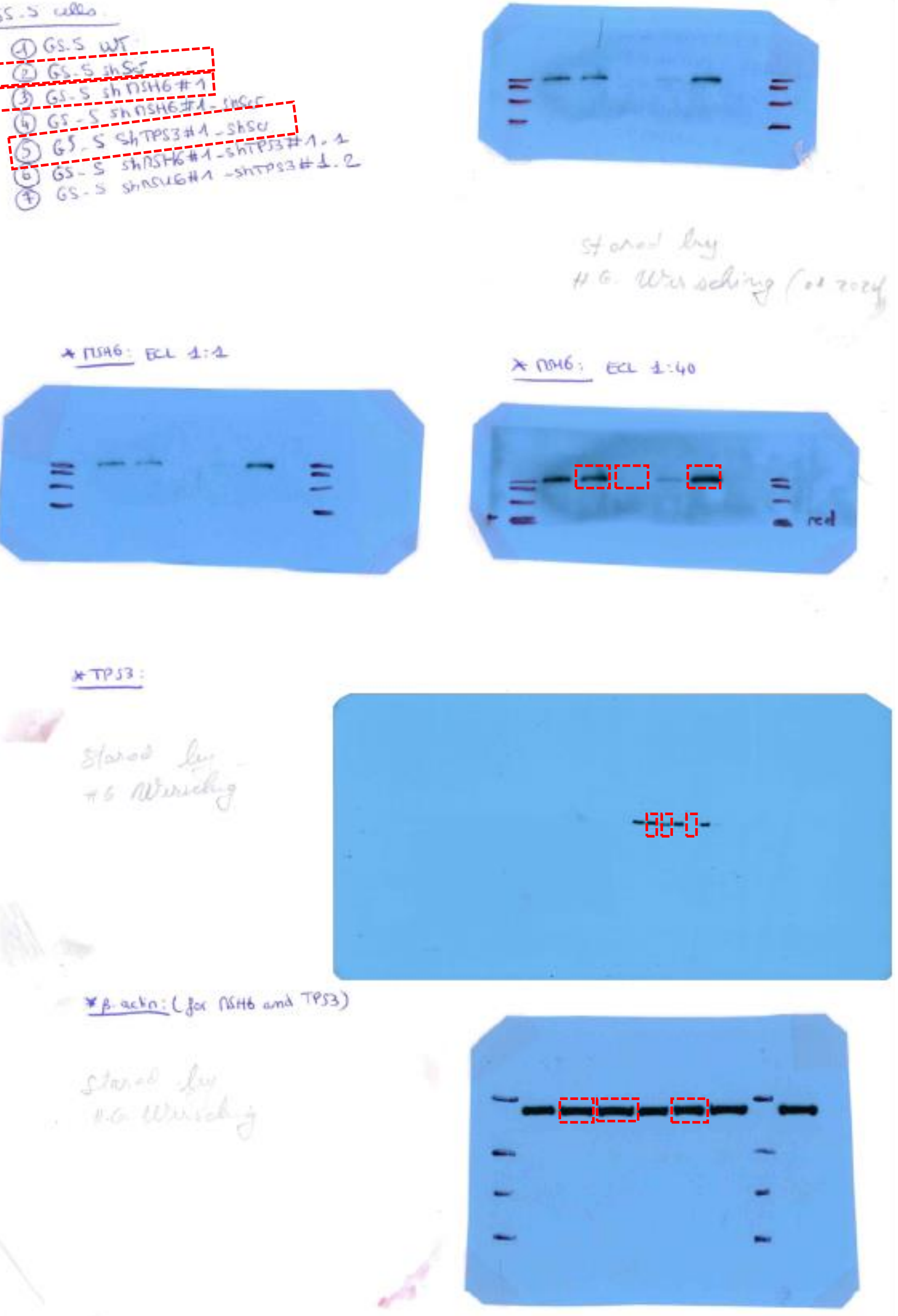

Raw western blots Figure S1F MSH6 & β actin

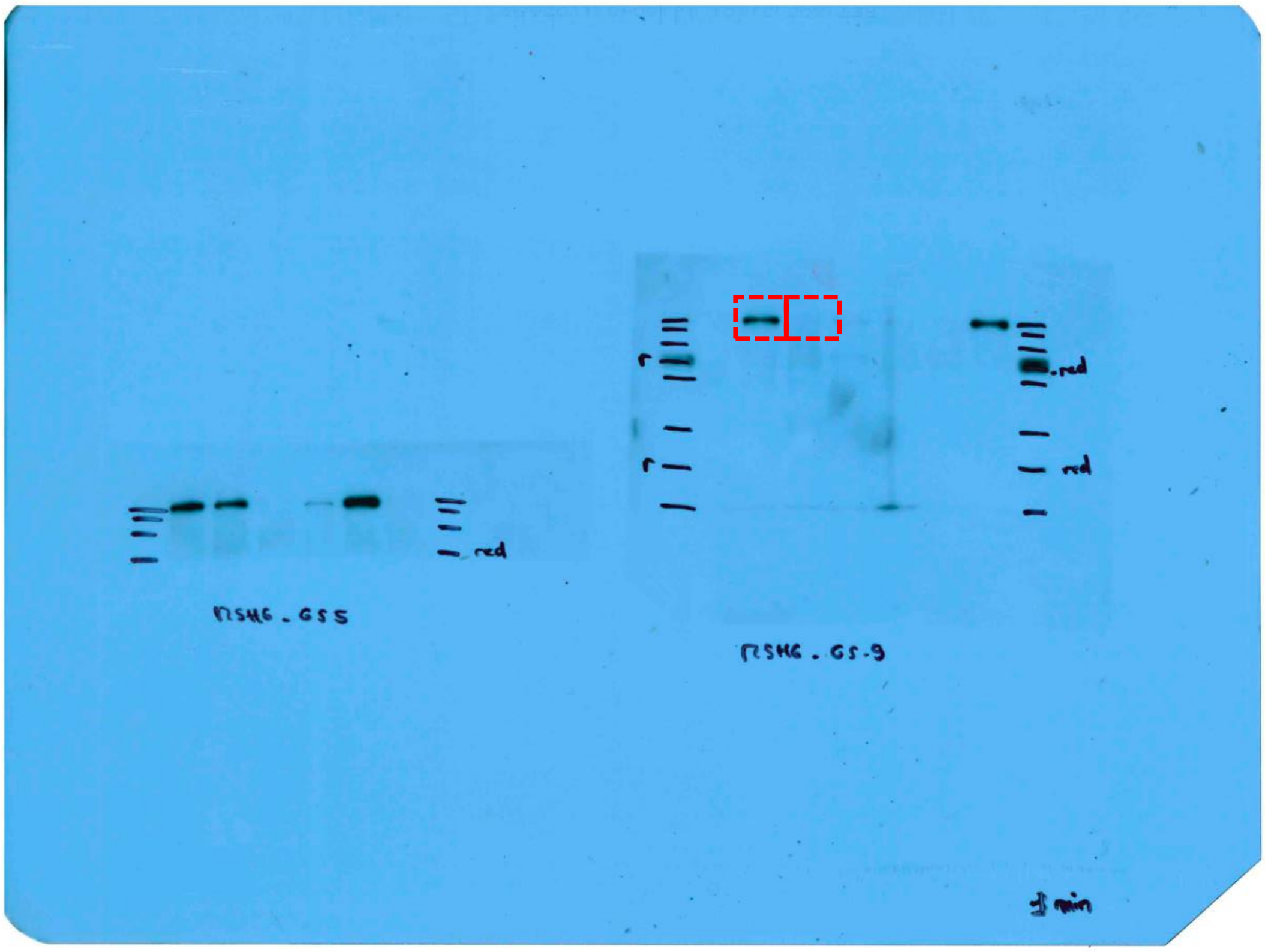

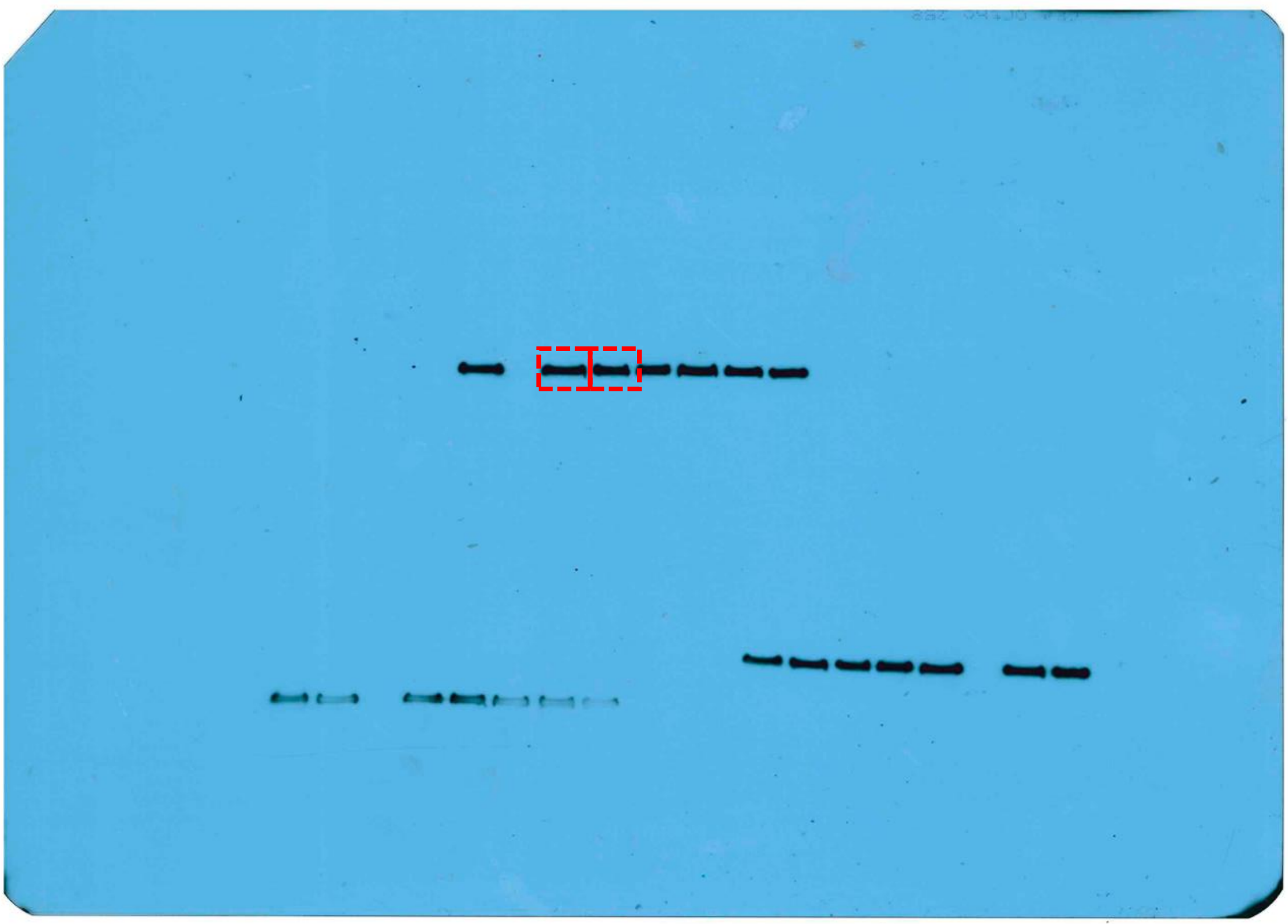

Raw western blots_Reporter Assay_Fig. S3c

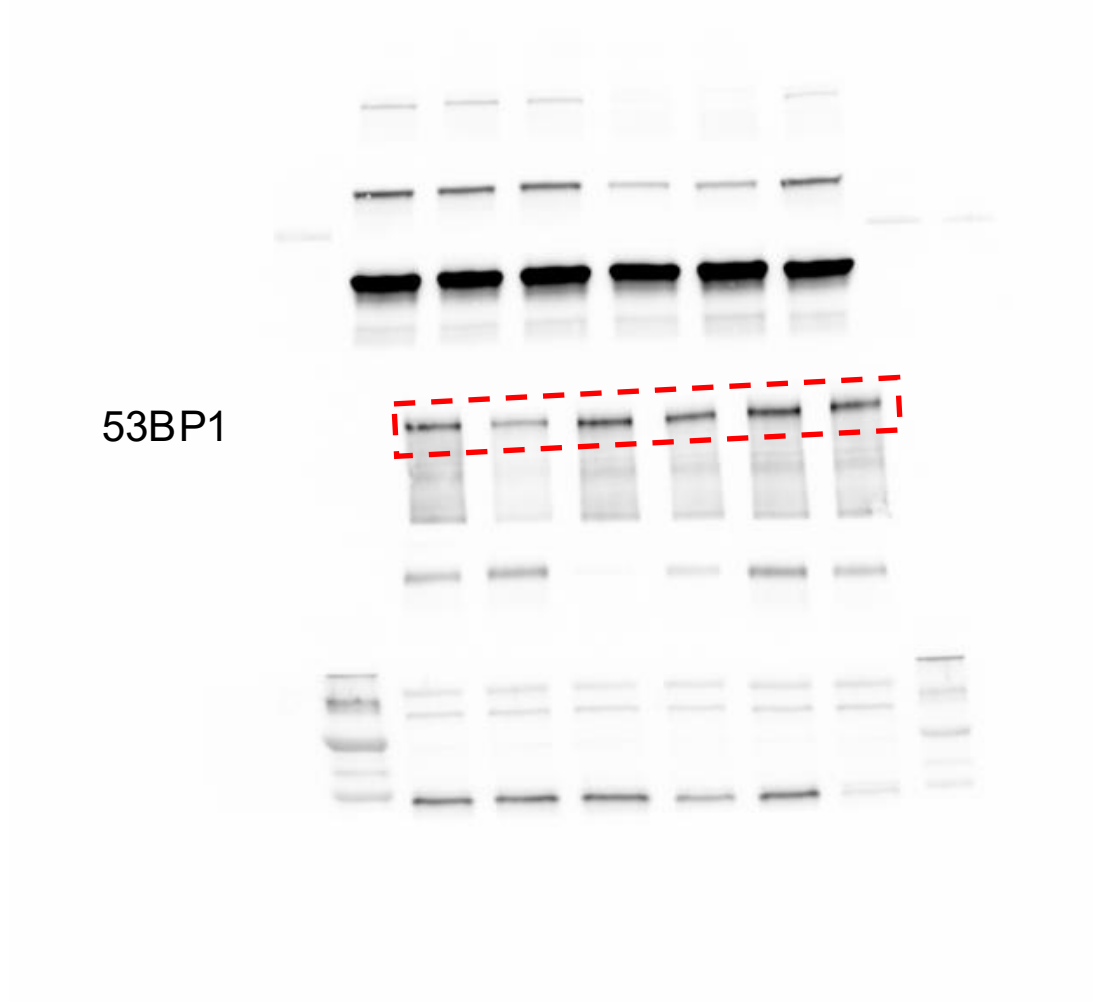

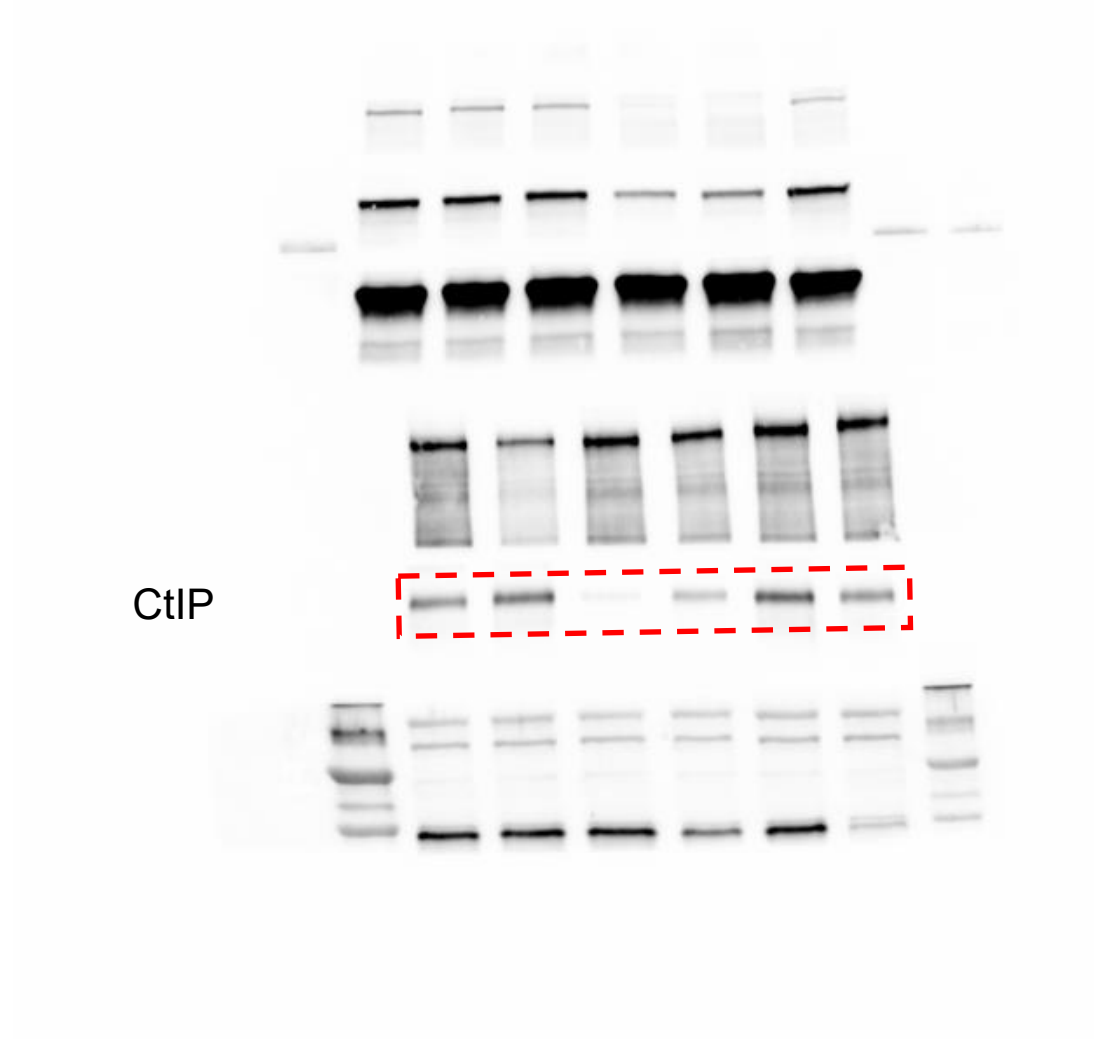

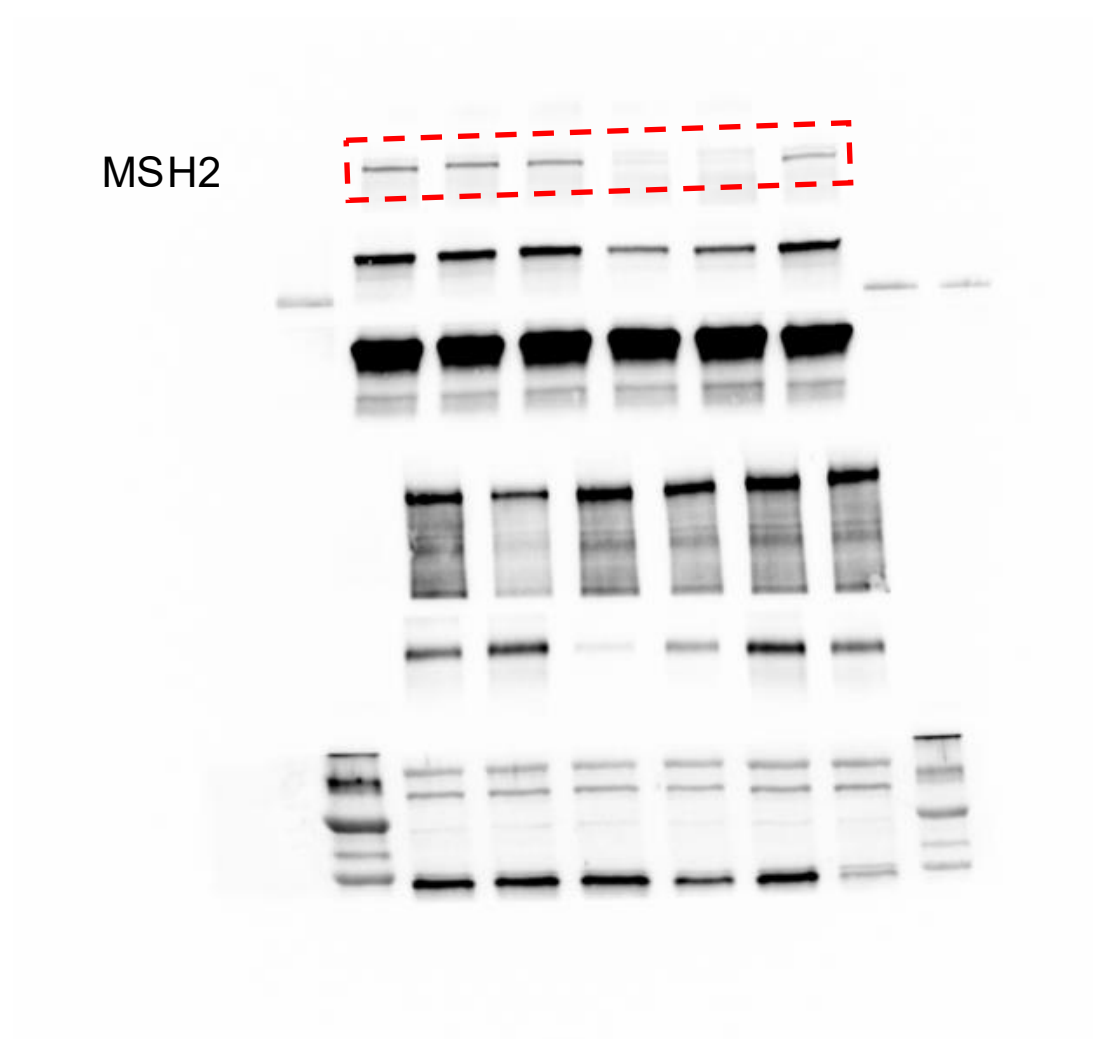

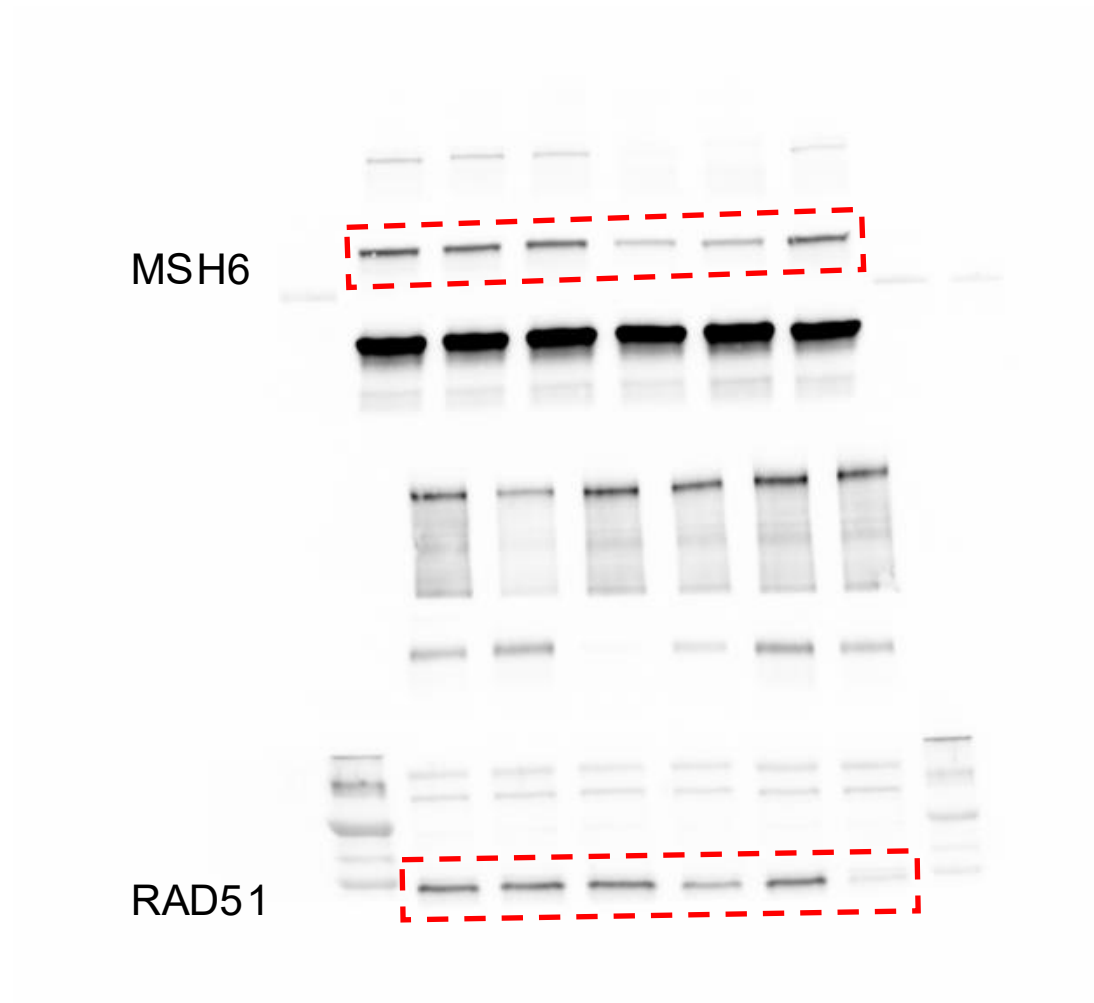

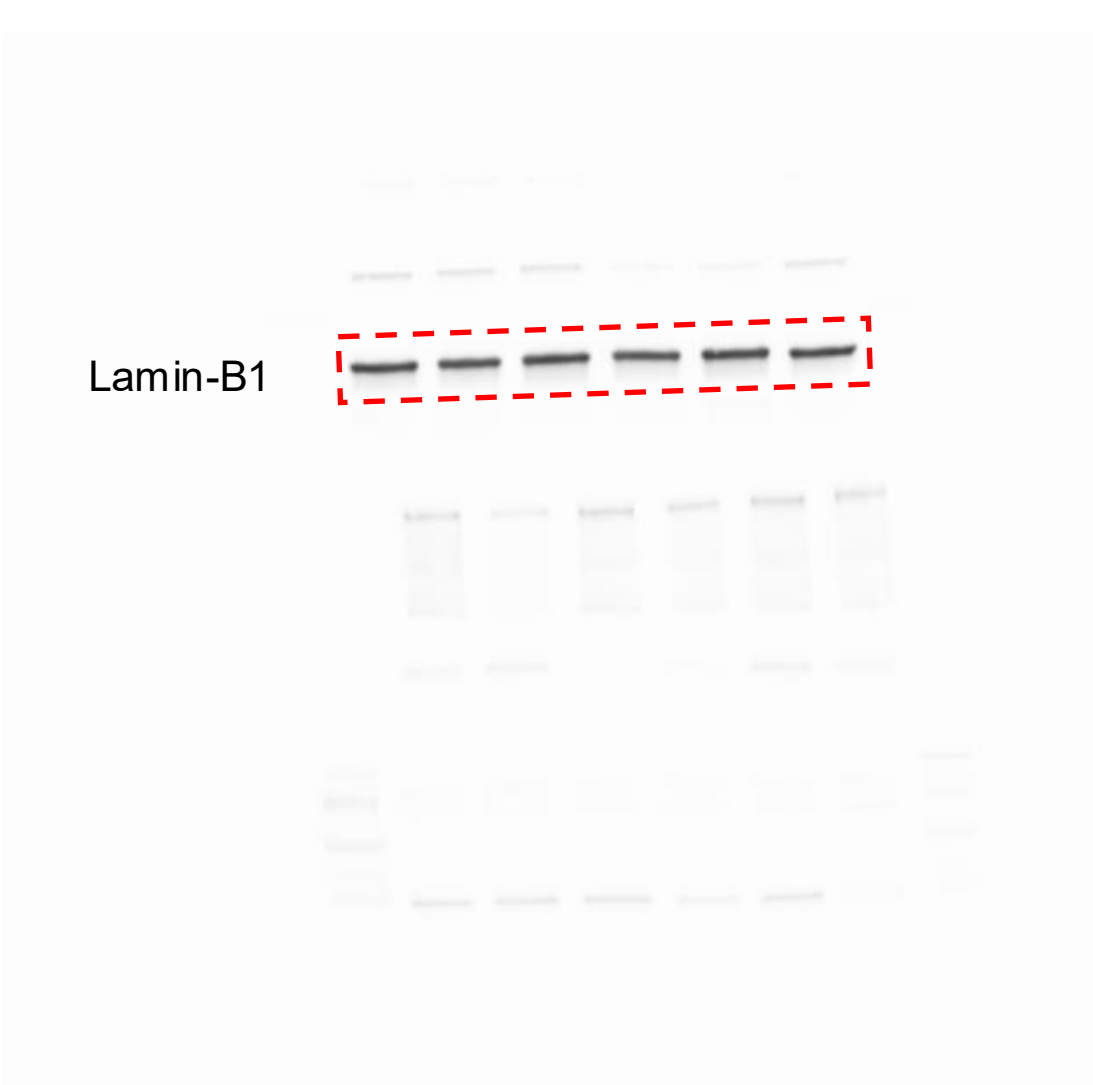

Raw western blots_MetaphaseSpreads_Fig. 1y

53BP1

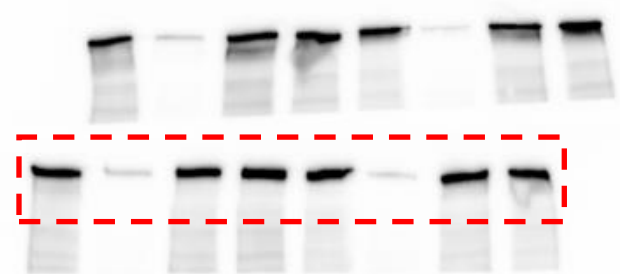

MSH2

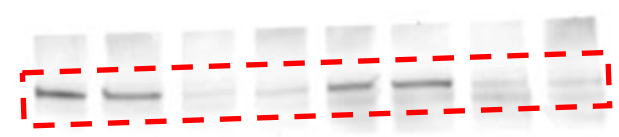

MSH6

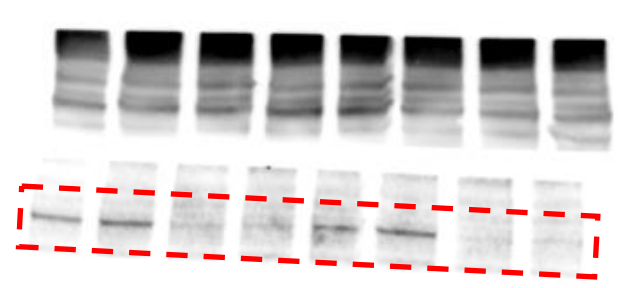

γH2AX

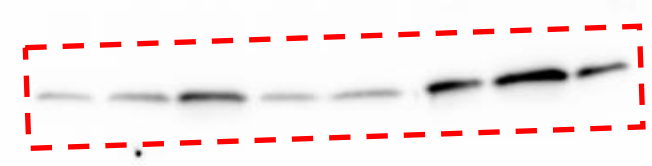

Lamin-B1

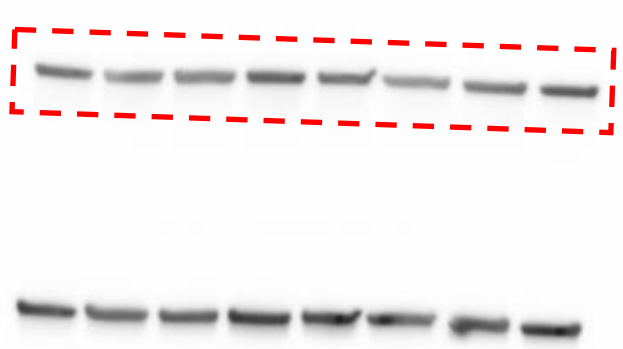

Supplement: Supplementary file 2 [file jci-136-205299-s217.pdf]
